# Blue‐Emitting ZnSe(Te) Quantum Dots and Light‐Emitting Diodes

**DOI:** 10.1002/advs.202600015

**Published:** 2026-05-29

**Authors:** Lijin Wang, Aiwei Tang, Jingbi You

**Affiliations:** ^1^ State Key Laboratory of Semiconductor Physics and Chip Technologies Institute of Semiconductors Chinese Academy of Sciences Beijing P. R. China; ^2^ Center of Materials Science and Optoelectronics Engineering University of Chinese Academy of Sciences Beijing P. R. China; ^3^ Key Laboratory of Luminescence and Optical Information Ministry of Education School of Physical Sciences and Engineering, Beijing Jiaotong University Beijing P. R. China

**Keywords:** blue emission, light‐emitting diodes, ZnSe(Te) quantum dots

## Abstract

Zn‐based quantum dots (QDs) have been regarded as the most promising Cd‐free candidates for blue‐emitting applications. ZnSe QDs exhibit an ultra‐narrow full width at half maximum (FWHM), high photoluminescence quantum yield (PLQY), and excellent environmental stability, making them ideal for blue‐violet emitters. ZnSe/ZnS core–shell structures have achieved a near 100% PLQY, thereby enabling high performance QDs light‐emitting diode (QLED) with an external quantum efficiency (EQE) of 13.6% at 443 nm. In order to extend the luminescence range for blue display, ZnSeTe QDs has been developed by doping Te ions into ZnSe. Currently, ZnSeTe QLEDs have achieved a maximum EQE of 24.7% at 460 nm, comparable to state‐of‐the‐art Cd‐based QLEDs, and a half‐lifetime of nearly 30 000 h (@100 cd/m^2^), demonstrating exceptional commercial potential. This review focuses on blue‐emitting ZnSe(Te) QDs, offering a comprehensive overview of recent advances. It presents a systematic discussion on ZnSe(Te) QDs, beginning with material properties and synthesis methods, following by the optimization strategies. Subsequently, the construction of LED devices is analyzed through improvements in both the emitting layer and carrier transport layers. Finally, the challenges in the development of ZnSe(Te) QLEDs are identified, and potential solutions are proposed, aiming to promote further advancements and accelerate their commercialization.

## Graphical abstract

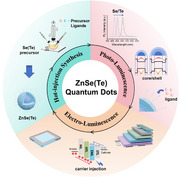

## 1 Introduction

Quantum dots (QDs) are nanocrystals (NCs) with sizes of 1–10 nm, exhibiting a significant quantum confinement effect. Louis E. Brus and Alexey I. Ekimov discovered the quantum confinement effect in the stained glass and synthesized QDs for the first time [1]. Building on these findings, Moungi G. Bawendi achieved near‐perfect CdSe QDs via a hot‐injection synthesis method, enabling the controllable synthesis of QDs in the laboratory [2]. Xiaogang Peng optimized the synthesis method and successfully realized a simple and high‐quality synthesis of CdE (E = S, Se, Te) QDs through a colloidal chemistry method, which paved the way for rapid development of QDs and laid the experimental foundation for further research [3]. Henceforth QDs have garnered considerable attention and experienced rapid development in the research field of displays [4], laser [5], biosensors [6], solar cells [7], and other semiconductor devices [8].

At present, Cd‐based QDs have been widely studied, and the lighting‐emitting diode (LED) exhibits outstanding properties [9]. But the performance of blue LED still falls short of the requirements for industrialization and urgently needs development [10]. After decades of research and exploration, the Blue QLED based on CdZnSe QDs exhibits a high external quantum efficiency (EQE) with 24.3% and a long operating T_95_ lifetime of over 150 h (@1000 cd/m^2^) [11, 12]. Although the Cd‐based materials exhibit outstanding properties, the biotoxicity of the Cd element limits further improvement in display application [13]. Therefore, several blue Cd‐free materials have been exploited and studied, such as II‐VI type Zn‐based QDs [14, 15], III‐V type InP QDs [16, 17], I‐III‐VI type NCs [18, 19], perovskite [20, 21], carbon dots [22, 23]. Blue‐emitting InP QDs require ultrasmall sizes due to the small bandgap, which cause synthesis difficulty and surface defects that lower fluorescence efficiency [24]. I‐III‐VI type NCs usually possess a wide full width at half maximum (FWHM) due to the defect‐related emission, which exhibits a low color purity [19]. Recently, I‐III‐VI type NCs with narrow FWHM have been developed, but their luminescence performance remains poor [25]. Perovskite materials have achieved ultra‐high EQE in the sky‐blue range (∼490 nm), but their performance remains relatively low in the pure‐blue (∼ 465 nm) and deep‐blue (∼ 450 nm) regions. Meanwhile, the lifetime of perovskite LEDs is much shorter than other QDs materials [26]. II‐VI type Zn‐based QDs have an appropriate bandgap for ideal blue emission range, narrow FWHM, excellent electroluminescence performance, and long service lifetime of LEDs. Therefore, II‐VI type Zn‐based QDs are widely regarded as the most promising blue‐emitting materials for replacing Cd‐based QDs and enabling industrial applications.

Zn‐based QDs exhibit a large bandgap and a composition similar to that of Cd‐based QDs, making them one of the most promising candidates for blue, cadmium‐free QDs. Specifically, ZnSe QDs demonstrate an ultra‐narrow FWHM, high PLQY, and excellent environmental stability, making them ideal emitters for blue‐violet light. Through extensive research efforts, ZnSe/ZnS core–shell structures have achieved a near‐100% PLQY, thereby enabling a high‐performance QLED device with an EQE of 13.6%. However, the latest Recommendation (Rec.) BT.2020 “Parameter Values for Ultra‐High‐Definition Television Systems for Production and International Programme Exchange” requires that the central wavelength for blue emission be 467 nm, with a FWHM of less than 20 nm [27]. The emission wavelength of ZnSe QDs is generally below 450 nm, which restricts their applicability in blue display applications. Although the emission peak can be shift to above 450 nm, the electroluminescence properties are usually dissatisfactory due to the large QDs size and Low carrier injection rate. To address this issue, researchers have developed ZnSeTe alloyed QDs by incorporating narrow‐bandgap ZnTe into the ZnSe lattice. By precisely tuning the anion Se/Te ratio, the emission peak is effectively red‐shifted into the desired blue region, enabling high‐efficiency blue LEDs that meet the standards of the display industry. The development of ZnSeTe‐based materials has been remarkable, with substantial progress achieved in less than a decade. Currently, ZnSeTe QLEDs have achieved a maximum EQE of 24.7%, comparable to state‐of‐the‐art Cd‐based QLEDs, and exhibit a half‐lifetime of nearly 30 000 h (@100 cd/m^2^), underscoring their exceptional potential for commercial applications. This review focus on the blue Zn‐based QDs, summarizing the development, including the synthesize, optimization, and surface passivation of Zn‐based QDs and the construction and optimization of Zn‐based QLEDs. It is providing references for researchers, with the aim of promoting the further development and commercial application process of Cd‐free QLEDs.

## 2 Bandgap of ZnSe(Te) QDs

**FIGURE 1:**
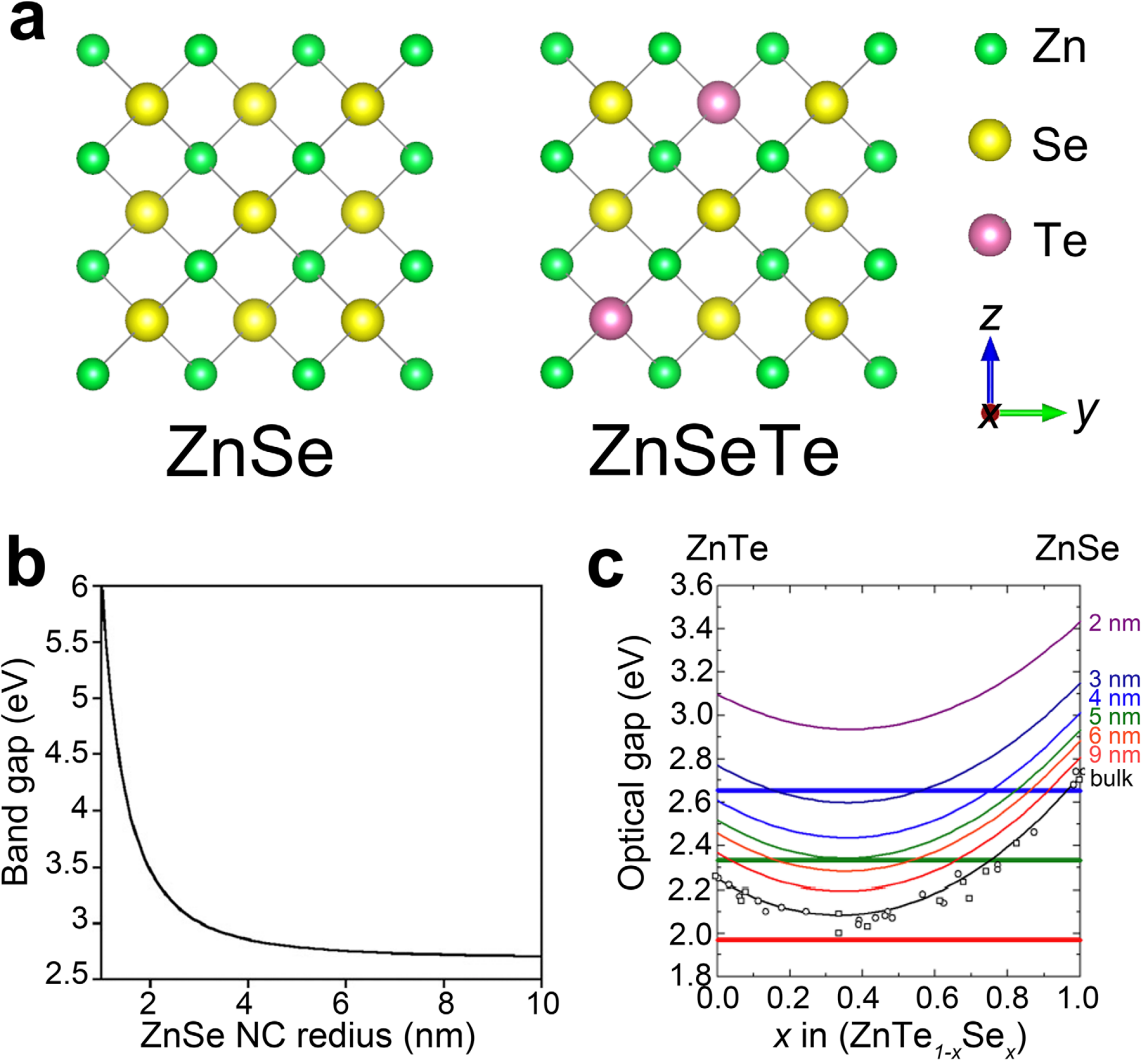
(a) The schematic diagram of the [100] crystal plane of the blende ZnSe(Te) crystal. (b) Correlation between the size of ZnSe NCs and their bandgap. Reproduced with permission from ref. [14]. Copyright 2007 Royal Society of Chemistry. (c) Composition dependence of the calculated optical gap of Zn(Te_1‐x_Se_x_) alloyed QDs. Reproduced with permission from ref. [30] under CC BY. Copyright 2017 AIP Publishing. Black open dots and lines indicate the composition dependence of the optical bandgap of bulk materials from refs. [31, 32, 33] for comparison.

Zn‐based QDs belong to the II‐VI type semiconductor NCs, similar to Cd‐based QDs. ZnSe(Te) QDs exhibit as a cubic blende structure. Figure 1a presents a schematic diagram of the ZnSe(Te) QD crystal, illustrating the [100] crystal plane. ZnSe materials, which are commonly used as blue‐violet emitters, have attracted significant research interest due to their bulk 2.7 eV bandgap and ultra‐narrow FWHM [14]. Figure 1b shows the relationship between the radius *r* of spherical ZnSe NCs and their bandgap, using the formula of Brus (*e*_1_) based on the effective mass approximation [28].

(1) $$E_{g}=E_{g}^{bulk}+\frac{\hbar^{2}\pi^{2}}{2r^{2}}\left(\frac{1}{m_{e}^{*}}+\frac{1}{m_{h}^{*}}\right)-\frac{1.8e^{2}}{4\pi \varepsilon \varepsilon_{0}}\frac{1}{r}$$

Due to the large bandgap of bulk ZnSe, the emission wavelength of ZnSe QDs generally below 450 nm. To extend the emission range, the first strategy is to increase the size of ZnSe QDs. The exciton Bohr radius of bulk ZnSe is approximately 4.5 nm, corresponding to a particle diameter of 9 nm [14]. When the particle diameter exceeds the Bohr radius, the strong confinement approximation (e.g., the Brus equation *e*_1_) is no longer strictly applicable, and the particles begin to exhibit properties more akin to those of bulk materials. However, due to the common passivation strategy using a type‐I core/shell structure (e.g., ZnSe/ZnS), the excitons remain confined within the nanocrystals, thereby preserving quantum confinement effects.

Another strategy is to dope ZnSe QDs with Te atoms to tune their bandgap. Utilizing the narrow bandgap of ZnTe (2.26 eV), ZnSeTe QDs formed by alloying ZnSe and ZnTe can effectively tune their bandgap through modulation of the Se/Te ratio, thereby extending the emission range of the QDs into the desired blue region. The composition‐dependent bandgap (*E_g_*) of bulk ZnSeTe solid solutions with a strong bandgap bowing is expressed as

(2) $$E_{g,ZnTe_{1-x}Se_{x}}=\left(1-x\right)E_{g,ZnTe}+xE_{g,ZnSe}-b_{ZnTe_{1-x}Se_{x}}\left(1-x\right)x$$

where x is the Se content in ZnTe_1‐x_Se_x_ and *b*_ZnTe1‐xSex_ is the bowing parameter with a value of 1.45 eV [29]. Both theoretical and experimental results have reported that ZnSeTe is a ternary alloy characterized by a large band‐bending constant. The band—anticrossing (BAC) model is the currently recognized explanation for band bending. In Te‐rich alloys, the band—anticrossing interaction between Se localized states and ZnTe conduction—band states lead to a reduction in the bandgap. In Se‐rich alloys, the minor substitution of Se by isoelectronic Te primarily causes a perturbation on the valence bands, forming a localized level above the valence band edge. Figure 1c depicts the energy band variation of bulk and nanocrystals ZnSe(Te) alloy. The blue, green, and red thick lines respectively indicate the light wavelengths recommended by BT.2000 (467, 532, and 630 nm, respectively) [30, 31, 32, 33]. Therefore, achieving the desired blue light emission requires precise control over both the QDs size and the alloying ratio. Selecting an appropriate preparation method is essential for the precise synthesis of QDs.

## 3 ZnSe(Te) QDs Synthesis Methods

**FIGURE 2:**
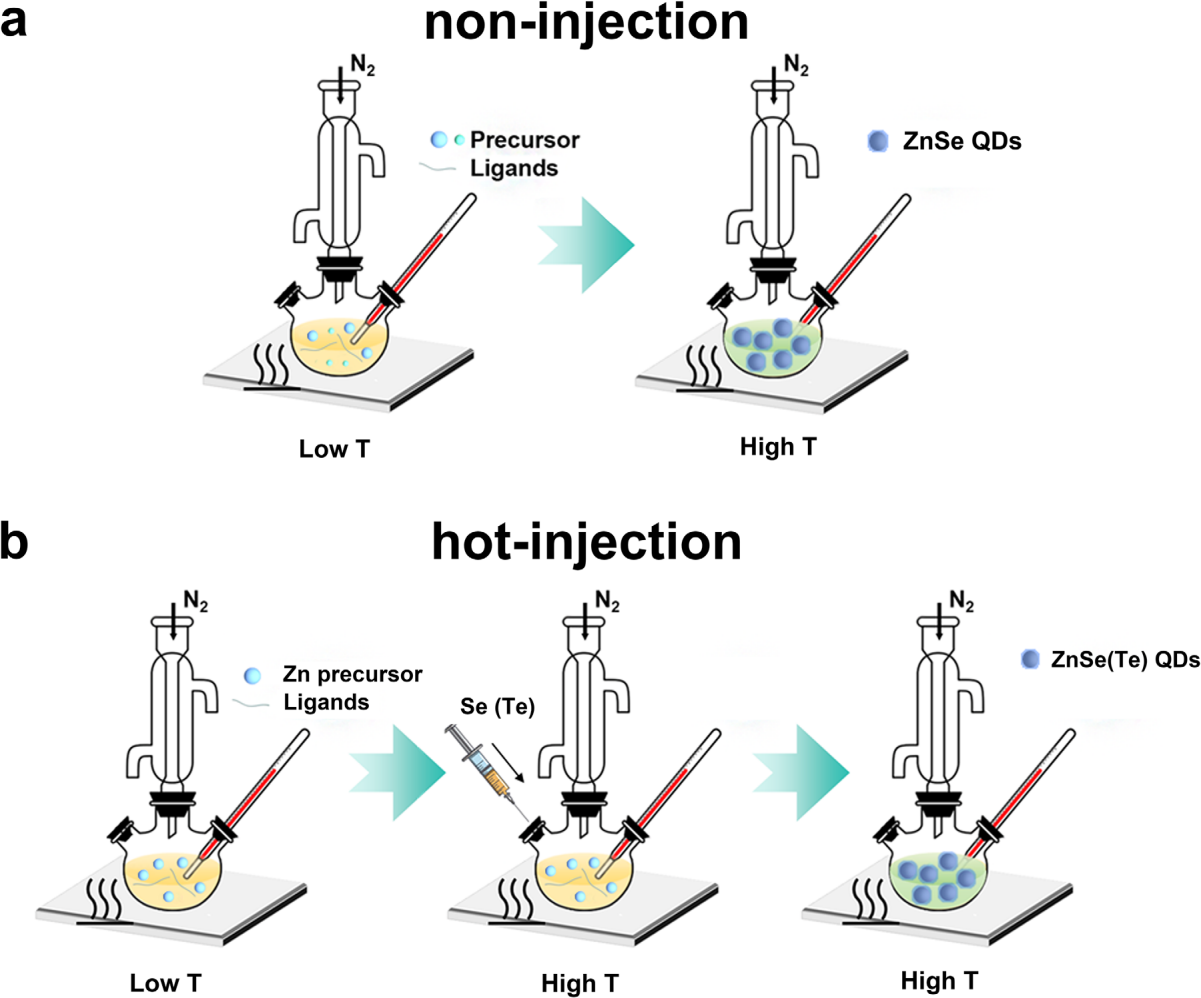
The schematic diagram of (a) the non‐injection method and (b) the hot‐injection method for ZnSe(Te) QDs.

In the early period, there are several methods to obtain ZnSe NCs, such as arrested precipitation [34], precipitation in the presence of stabilizers [35, 36, 37], and synthesis in reverse micelles [38, 39, 40, 41]. But the QDs obtained through these complex methods generally perform a low PLQY and poor stability. High‐quality colloidal ZnSe QDs were first reported in 1998 by Hines et al., resulting from the organometallic reaction of organometal diethylzinc (Et_2_Zn) and Se powder in a hexadecylamine/ trioctylphosphine (HDA/TOP) coordination solvent [42]. This colloidal synthesis method in the organic phase can refrain from the issues such as low crystallization, uneven size distribution, low PLQY, and poor stability from the aqueous phase system. The mainstream synthesis methods for ZnSe(Te) QDs mainly include the non‐injection and hot‐injection method. (Figure 2).

### 3.1 Non‐Injection Method

Non‐injection method, also known as the heating‐up method, involves introducing all precursors into the reaction system at the outset and subsequently generating QDs through controlled heating at a specific temperature. This method has the advantages of simple operation and easy scalability, and has been widely used in the large‐scale synthesis of metal and oxide nanoparticles.

Cumberland et al. synthesized 2–5 nm ZnSe QDs employing this method for the first time by adopting air‐stable [Zn_10_Se_4_(SPh)_16_]^4^^−^ clusters as monomolecular precursors in HAD, with particle size controlled by varying the reaction temperature between 220°C and 280°C [43]. Liu et al. directly mixed zinc acetate, selenium powder, oleic acid, and paraffin oil, followed by heating to prepare ZnSe QDs [44]. The size, morphology, and crystalline phase of the QDs can be effectively controlled by modulating experimental parameters, including precursor concentration, the Zn/Se molar ratio, and the heating rate. Nevertheless, the PLQY of the QDs remains relatively low, below 15%.

Although the non‐injection method can be used in large‐scale production of QDs, it lies on precursor activity and temperature. The nucleation in the certain time of increase temperature process is difficult to control, which increases the complexity of the synthesis process and hinders precise control over QDs growth, as all precursors react simultaneously within the same system. Furthermore, the shell growth process also cannot be realized through a non‐injection method.

### 3.2 Hot‐Injection Method

The hot‐injection method is a typical method to obtain QDs. The hot‐injection method refers to a rapid process of injection the precursor into the synthesis system at a certain temperature. The rapid injection of precursor causes monomer oversaturation, resulting in a rapid nucleation explosion. Whereafter nucleation is inhibited with the decrease of monomer concentration, thus promoting the growth of QDs. For ZnSe QDs, the precursor of Zn and Se are injected into a hot reaction system or injecting one precursor into the thermal system containing other one precursor. While for ZnSeTe QDs, almost all reported researches adopted hot‐injection, in which the Se and Te precursor was rapidly injected into a hot thermal system containing Zn precursor.

In early studies focused on the synthesis of ZnSe, the solvent‐ligand combination was employed. For instance, a monomolecular precursor, ethyl(diethyldiselenocarbamato)zinc, was dissolved in tri‐n‐octylphosphine (TOP) and injected into hot tri‐n‐octylphosphine oxide (TOPO), yielding nanocrystals with a relatively broad emission peak centered at 446 nm [45]. Based on this approach, Hines and Guyon–Sionnest synthesized ZnSe QDs exhibiting strong band‐edge emission in the 365–445 nm range, with PLQYs of 20–50%, using organometallic precursors diethyl zinc and TOP‐Se injecting into hot Hexadecylamine (HDA) [42]. HDA was employed to eliminate the dangling bonds in the surface trap states of QDs, thereby achieving pure edge‐state fluorescence.

At present, the toxicity and highly flammable Zn precursor, diethyl zinc was replaced by stability Zn sources, such as Zinc stearate (Zn(St)_2_), Zinc acetate (Zn(OAc)_2_), and ZnO. Reiss and his group optimized the synthesis strategy by replacing flammable diethyl zinc with gas‐phase stable zinc stearate [46], Zinc stearate functions not only as a cationic precursor but also as a stabilizing agent for stearic acid ligands. By precisely controlling the precursor concentration and reaction temperature, ZnSe QDs exhibited a tunable size of 3–7 nm and luminescence peak of 394–440 nm.

The coordination solvents (e.g. HAD, TOPO) used in the reaction can not only control the growth rate of QDs, but also passivate the surface. However, the suitable solvents are limited, and it is difficult to control the nucleation and growth. Therefore, non‐coordination solvent/ligand combinations have been developed. The typical non‐coordination including n‐octadecane, 1‐octadecene (ODE), natural paraffin oil, trioctylamine (TOA), and so on. For instance, Shen et al., inspired by Peng's synthetic approach to CdSe, further simplified the reaction process by replacing the coordinating solvents HDA and TOPO with the non‐coordinating solvent ODE [47]. Uniform ZnSe QDs were synthesized without the use of organic phosphines or fatty amines. By controlling the reaction temperature, QDs exhibiting photoluminescence (PL) peaks ranging from 390 to 450 nm with the FWHM between 14 and 17 nm were obtained. Moreover, the PLQY of QDs exceeded 70% after coating the QDs with a ZnS shell.

For anion precursor, organic phosphines such as TOP and diphenylphosphine (DPP), which are both ligands and solvents, can coordinate with anion (Se and Te), whose reactivity affects the reaction kinetics and plays a key role in ZnSe(Te) QDs. The Yang group successfully prepared ternary ZnSeTe QDs via the hot‐injection method. The anion precursor Te‐TOP was injected into the ODE solvent that dissolved the Zn and Se precursors. The emission spectrum could be tuned within the range of 422–500 nm by adjusting the Se/Te ratio [48]. Following the deposition of ZnSe and ZnS shells, the PLQY of the resulting QDs reached 70%, with a FWHM of 32 nm. Subsequently, Kim et al. employed the Se‐TOP, Te‐TOP and DPP as the anion precursor, the mixture was injection into the Zn precursor to obtain the ZnSeTe core QDs [49]. Following shell coating, the obtained ZnSeTe/ZnSe/ZnS QDs exhibited a PL peak at 457 nm with a PLQY approaching 100%.

## 4 Luminescence Properties Optimization of ZnSe(Te) QDs

After extensive investigation, blue‐emitting ZnSe(Te) QDs have exhibited excellent properties of high PLQY (∼ 100%). However, ZnSe(Te) QDs also exhibit several shortcomings as ideal blue‐emitting materials. The intrinsic emission of ZnSe QDs can be narrowed below 10 nm, while the wavelength commonly locates below 450 nm beyond the requirements for blue light display. The alloyed ZnSeTe QDs exhibit a broad FWHM (> 30 nm) due to the uneven ion doping, which leads to a relatively poor color purity. In order to obtain the high‐quality ZnSe(Te) QDs, several strategies are researched to improve the QDs luminescence properties in previous research works. Following, we will summarize these strategies from several aspects.

### 4.1 Particle Size

**FIGURE 3:**
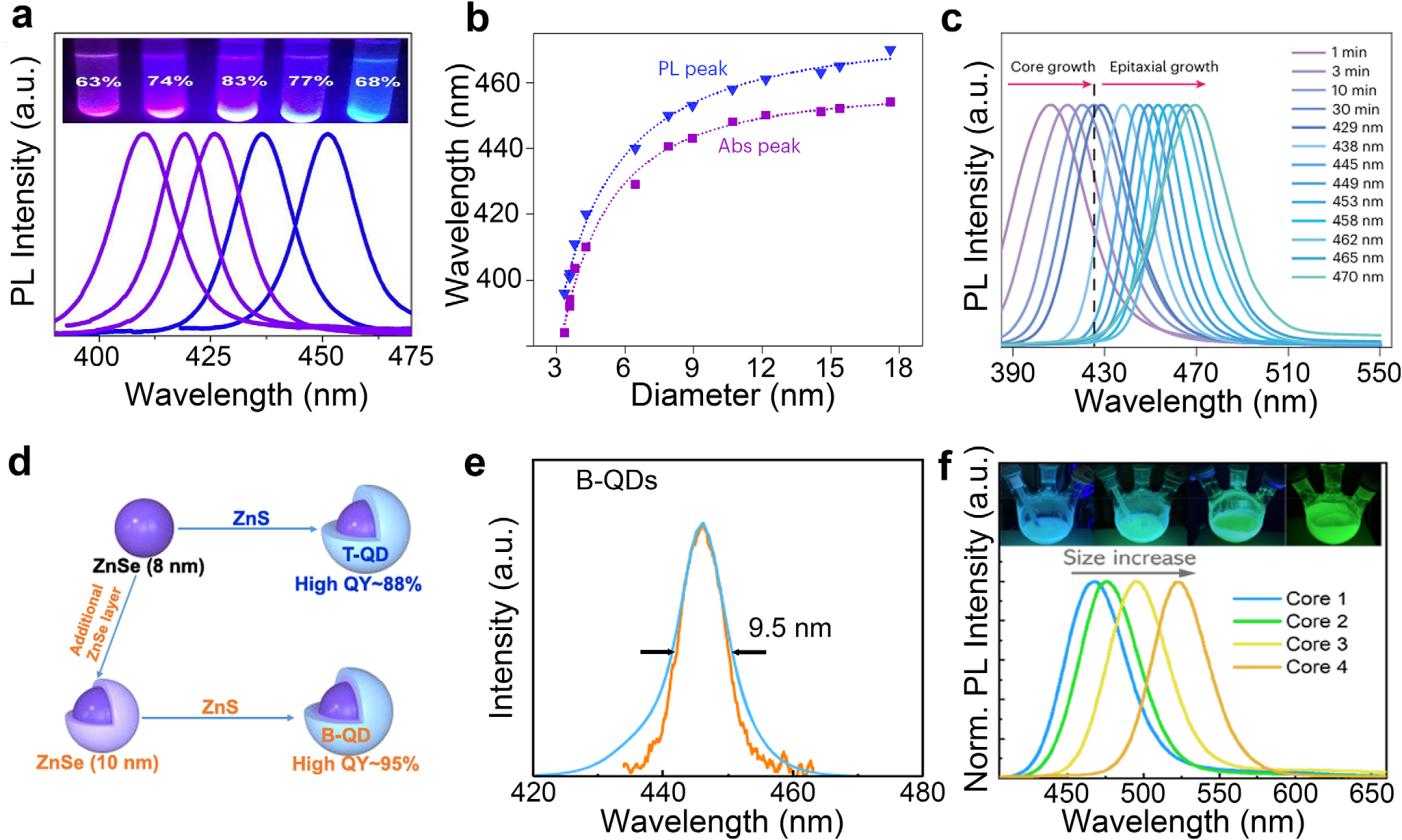
(a) Normalized PL spectra of ZnSe/ZnS core/shell QDs starting from different ZnSe cores, and representative emission colors of ZnSe/ZnS core/shell QDs under the radiation of a UV lamp. Reproduced with permission from ref. [50]. Copyright 2015 Royal Society of Chemistry. (b) The PL and Abs peaks versus the diameter (D) of ZnSe nanocrystals. (c) PL spectra of ZnSe nanocrystals during the RCEG process. Reproduced with permission from ref. [52]. Copyright 2023 Springer Nature. d) Schematic illustration of the controlled synthesis of two types of ZnSe/ZnS core/shell QDs. Traditional ZnSe/ZnS QDs (T‐QDs) were obtained by growing a ZnS shell (4 MLs) on the ∼8.0 nm ZnSe core. Bulk‐like ZnSe/ZnS QDs (B‐QDs) were obtained by growing a ZnS shell (4 MLs) on the ∼10.0 nm ZnSe core. (e) Spectral correlations of the ensemble (cyan) and the single‐QD PL spectra (orange) of B‐QDs. Reproduced with permission from ref. [53]. Copyright 2021 American Chemical Society. (f) Normalized PL spectra of different‐sized ZnSeTe cores synthesized with the same feeding ratio of Se/Te = 4. Reproduced with permission from ref. [54]. Copyright 2023 American Chemical Society.

According to the quantum confinement effect, the PL peak position of QDs can be tuned through controlling the particle size, as mentioned above in Section 2. As discussed above, the quantum confinement effect weakens as the particle size increases to approach or exceed the exciton Bohr radius. Therefore, describing such materials as QDs introduces ambiguity. Instead, the term NCs should be used for large particles. Wang et al. have already certified that the larger the nucleus size of ZnSe NCs, the more obvious the spectral redshift, and displaying the emission color gradually changing from purple to blue [50]. Figure 3a presents the normalized PL spectra and emission color evolution of ZnSe/ZnS QDs with different core sizes. Yang et al. synthesized ZnSe core QDs with a particle size from 5.3 to 12.2 nm by sequentially adding Zn and Se precursor solution on ZnSe NCs [51]. The obtained ZnSe/ZnS NCs exhibited a PL peak redshift from 422 to 443 nm after further ZnS shell coating.

Typically, the emission wavelength of ZnSe QDs below 450 nm, which restricts their applicability in blue display applications. Experimental results of PL and absorbance peaks versus the diameter (D) of ZnSe NCs as shown in Figure 3b [52]. The measured PL peak positions can be fitted using a power law relation of λ_PL_ = 476.02 × D^1.42^/(1.08^1.42^ + D^1.42^). It can be predicted that the ultra‐large ZnSe core (> 17 nm) can obtain a blue emission wavelength of 450 nm. Consequently, synthesizing larger‐sized ZnSe NCs is essential to align them with the desired blue‐emitting range in display technologies.

Zhong et al. developed a nuclei number‐considered LaMer model to illustrate the correlation between the final size of nanocrystals and the total number of nuclei [52]. A general strategy for reactivity‐controlled epitaxial growth (RCEG) has been proposed to enable the synthesis of large‐diameter ZnSe NCs with an average size exceeding 35 nm. Large‐diameter ZnSe NCs were prepared by successively injecting high‐activity and low‐activity Zn and Se precursors for core growth and epitaxial growth, respectively. The obtained large‐diameter ZnSe NCs exhibited pure blue fluorescence in the wavelength range of 455–470 nm, with a FWHM of 16–25 nm. (Figure 3c) After coating the ZnS shell, the obtained spherical ZnSe/ZnS NCs exhibited a PLQY of 60%. The Shen group further utilized an epitaxial growth strategy to extend the deposition of ZnSe onto pre‐formed ZnSe QDs nuclei, resulting in large‐sized (∼10 nm), bulk‐like ZnSe structures [53]. Subsequently, a ZnS shell was deposited to fabricate ZnSe/ZnS core/shell NCs exhibiting a PL peak at 445 nm, a PLQY of 95%, and a narrow FWHM of 9.6 nm (Figure 3d,e).

For ZnSeTe QDs, the particle size can also be tuned by controlling the reaction temperature. small‐ (core 1), medium‐ (core 2), and large‐sized ZnSeTe (core 3) were synthesized by adjusting the reaction temperature at 240°C, 270°C, and 300°C, respectively [54]. The size was further increased by injecting Zn, Se, Te stock solutions to obtain core 4. Figure 3f illustrates that the emission wavelength of these ZnSeTe cores is significantly redshifted from 469 nm to the green region of 521 nm.

### 4.2 Alloying Strategy

**FIGURE 4:**
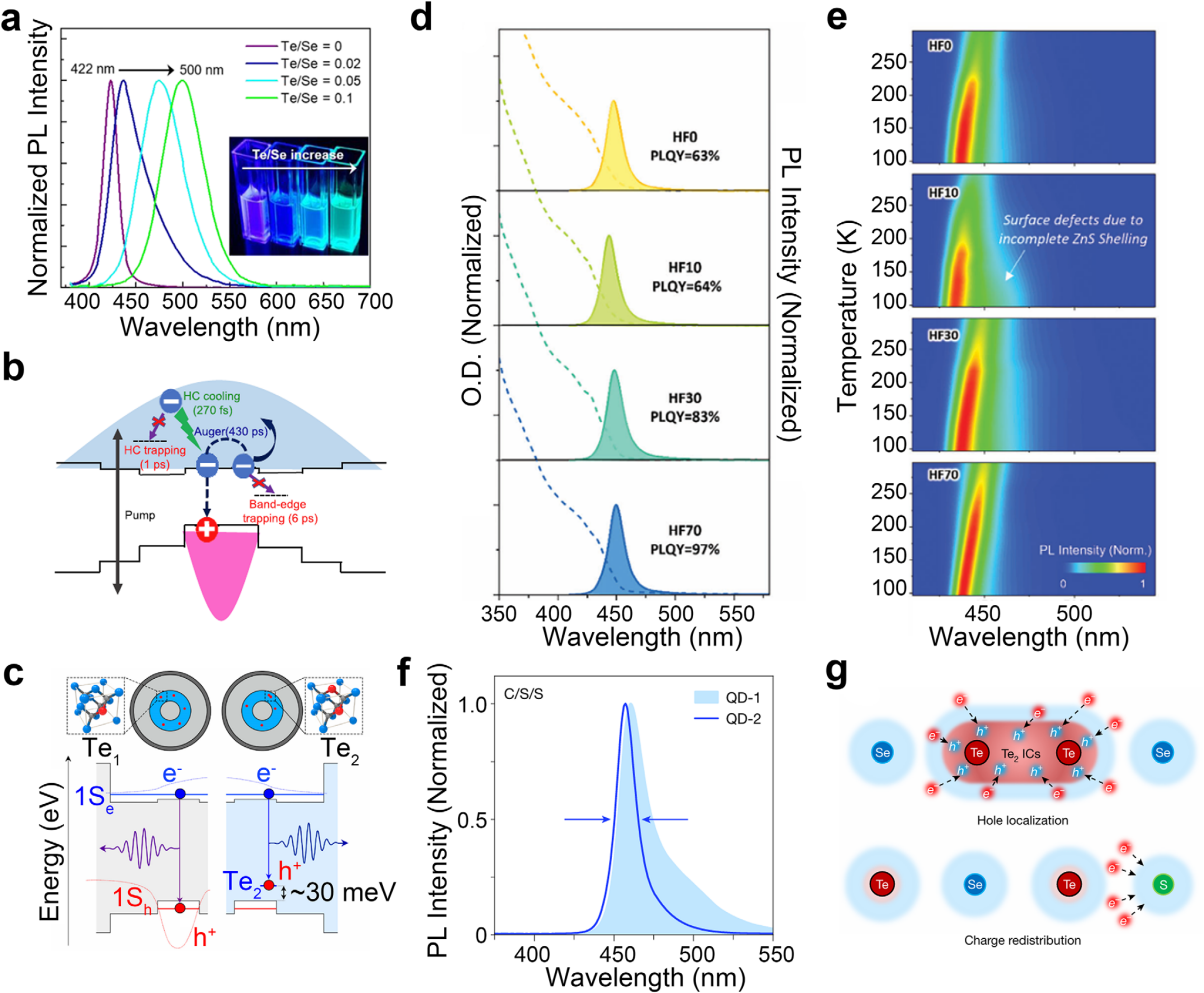
(a) Normalized PL spectra and fluorescent images (inset) of ZnSeTe/ZnS core/shell QDs as a function of Te/Se molar ratio. Reproduced with permission from ref. [48]. Copyright 2019 American Chemical Society. (b) Schematic depiction of the energy band of ZnSeTe/ZnSe/ZnSeS/ZnS CQDs with a quasi‐type II structure and the carrier dynamic processes, including electrons trapping by defect states, hot carrier (HC) cooling, and Auger recombination. Reproduced with permission from ref. [56]. Copyright 2021 American Chemical Society. (c) Band structure and electronic energy levels of ZnSe/ ZnTe_x_Se_1‐x_/ZnSe/ZnS NCs without (left) vs with the nearest‐neighbor pair of Te atoms. Reproduced with permission from ref. [57]. Copyright 2022 American Chemical Society. (d) Steady‐state absorption (dashed line) and PL spectra (solid line) and (e) Temperature‐dependent 2D PL spectra, at 97–297 K, of HF0, HF10, HF30, and HF70 QDs. Reproduced with permission from ref. [59]. Copyright 2024 John Wiley and Sons. (f) PL spectra of C/S/S QD‐1 (ZnSeTe/ZnSe/ZnS) and QD‐2 (ZnSeTeS/ZnSe/ZnS). (g) Schematic illustration of charge redistribution in the Te and S model. ICs, isoelectronic centres. Reproduced with permission from ref. [60]. Copyright 2025 Springer Nature.

Although increasing the particle size enables ZnSe QDs to achieve deep‐blue emission, the resulting large‐sized QDs show reduced PLQY. Therefore, researchers developed an alloying strategy that introduces Te into ZnSe QDs, resulting in alloyed ZnSeTe QDs with ideal blue emission. The Yang group first obtained ZnSeTe/ZnS QDs with emission wavelengths ranging from 422 to 500 nm by adjusting the Se/Te ratio. (Figure 4a) [48]. Subsequently, similar results have been widely reported.

Although the ionic radius of Te (2.21 Å) is larger than that of Se (1.98 Å), Te ions can still be successfully incorporated into the ZnSeTe QDs. Nevertheless, as observed in the PL spectrum, with the increase of Te doping content, it always leads to an asymmetric broadening of the PL spectrum, primarily manifested as a low‐energy tail emission. The origin of this spectral asymmetry remains unclear at present. Two primary hypotheses have been proposed by researchers to explain this phenomenon:

- The first hypothesis posits that the observed phenomenon is associated with lattice defects resulting from the significant difference in atomic radius between Se and Te, which may give rise to shallow trap states situated above the valence band maximum [55]. Huang et al. for the first time decoded the ultrafast hot carrier trapping (<2 ps) and band‐edge carrier trapping processes (∼6 ps) in the ZnSe QDs system [56], which was determined to be the main culprit for the deteriorated PLQY in previous reports. (Figure 4b) In fact, owing to the considerable discrepancy between Se^2−^ and Te^2−^ ions in a ternary ZnSeTe compound, it becomes more probable that many intrinsic and doping‐related defects would be generated and rapidly capture photoexcited electrons, resulting in poor PLQY.
- The second hypothesis posits that the significant difference in electronegativity between Te and Se induces the formation of localized hole states when Te ions act as nearest‐neighbor pairs [57]. Consequently, the observed phenomenon may be attributed to spatially separated excitons. These excitons feature delocalized electrons and localized holes, and are confined within Te clusters (Te_n≥2_), which serve as trapping sites. (Figure 4c) Imran et al. observed that clustering of the Te atoms in the ZnSe lattice induces an upshift of the highest occupied molecular orbital (HOMO) energy level while maintaining the lowest unoccupied molecular orbital (LUMO) energy level. An increase in the size of the Te cluster results in a larger upshift of the HOMO level as well as the appearance of more discrete energy states near the HOMO level. Such localization of states near the HOMO level causes spectral broadening of Te‐alloyed QDs.

At present, the researchers have not reached a consensus for these two hypotheses. To eliminate this PL spectra broadening phenomenon, current research efforts are predominantly focused on controlling interstitial defects and achieving a homogeneous distribution of Te atoms. Imran et al. developed a synthetic strategy employing benzoyl fluoride (BF) as an additive to achieve uniform distribution of Te atoms within the crystal lattice during the nucleation and growth stages, resulting in a reduction of the FWHM from 36 to 22 nm [58]. BF reacts sequentially with surfactants, resulting in the formation of intermediate F···H···trioctylamine adducts that function as a pseudo‐HF source by releasing anhydrous HF. The controlled release of HF during nucleation and growth stages homogenizes distribution of Te within ZnSeTe lattice, resulting in a narrow FWHM. Subsequently, Lee et al. synthesized blue‐emitting ZnSeTe QDs with a PLQY of 97% by employing an excess of HF additive, achieving an FWHM of merely 14 nm [59]. (Figure 4d,e) Sufficient HF concentration effectively inhibited photobleaching, leading to a stable blinking process in the QDs. The facet‐specific growth of the QDs facilitated a monodisperse morphology, thereby enhancing spectral uniformity.

Recently, the Yang group developed an isoelectronic control strategy that utilizes a highly electronegative homologous carrier S to interfere with the surrounding charge carrier environment [60]. This approach effectively mitigates the hole localization associated with Te atoms, thereby inhibiting the formation of Te_n_ (n≥2) sub‐centers, enhancing structural stability, and reducing the density of non‐radiative recombination centers. This strategy effectively narrowed the FWHM of QDs and enhanced the radiative recombination efficiency. As a result, the synthesized ZnSeTeS/ZnSe/ZnS QDs exhibit a near‐100% PLQY at 460 nm, with a FWHM of only 16.8 nm. (Figure 4f,g).

### 4.3 Surface Etching of Core

Bare ZnSe(Te) cores generally exhibit poor stability. Upon purification and removal from the original synthesis system, their surfaces are susceptible to the formation of oxidation states and various structural defects, which can substantially degrade interfacial integrity. A common strategy to address this issue is to remove the oxide species from the core surface via a strong acid etching process, thereby facilitating subsequent shell growth.

In 2019, the research groups of Jang and Kim collaborated to utilize hydrofluoric acid (HF) for etching InP cores, achieving near‐100% PLQY and a device efficiency of up to 21.4% in InP/ZnSe/ZnS QDs [61]. However, the application of HF raises significant concerns due to its high corrosivity and toxicity, as well as safety risks associated with the rapid pressure increase during precursor injection at elevated temperatures. He et al. proposed an alternative approach involving the introduction of ammonium fluoride (NH_4_F) at the early stage of synthesis, thereby eliminating the need for hazardous HF treatment [62]. The fluoride ions (F^−^) released by NH_4_F serve a function analogous to that of HF in the reaction. As a result, the as‐prepared ZnSeTe/ZnSe/ZnS QDs exhibit continuously tunable emission wavelengths within the pure blue region (450–465 nm), achieving a PLQY as high as 95%.

**FIGURE 5:**
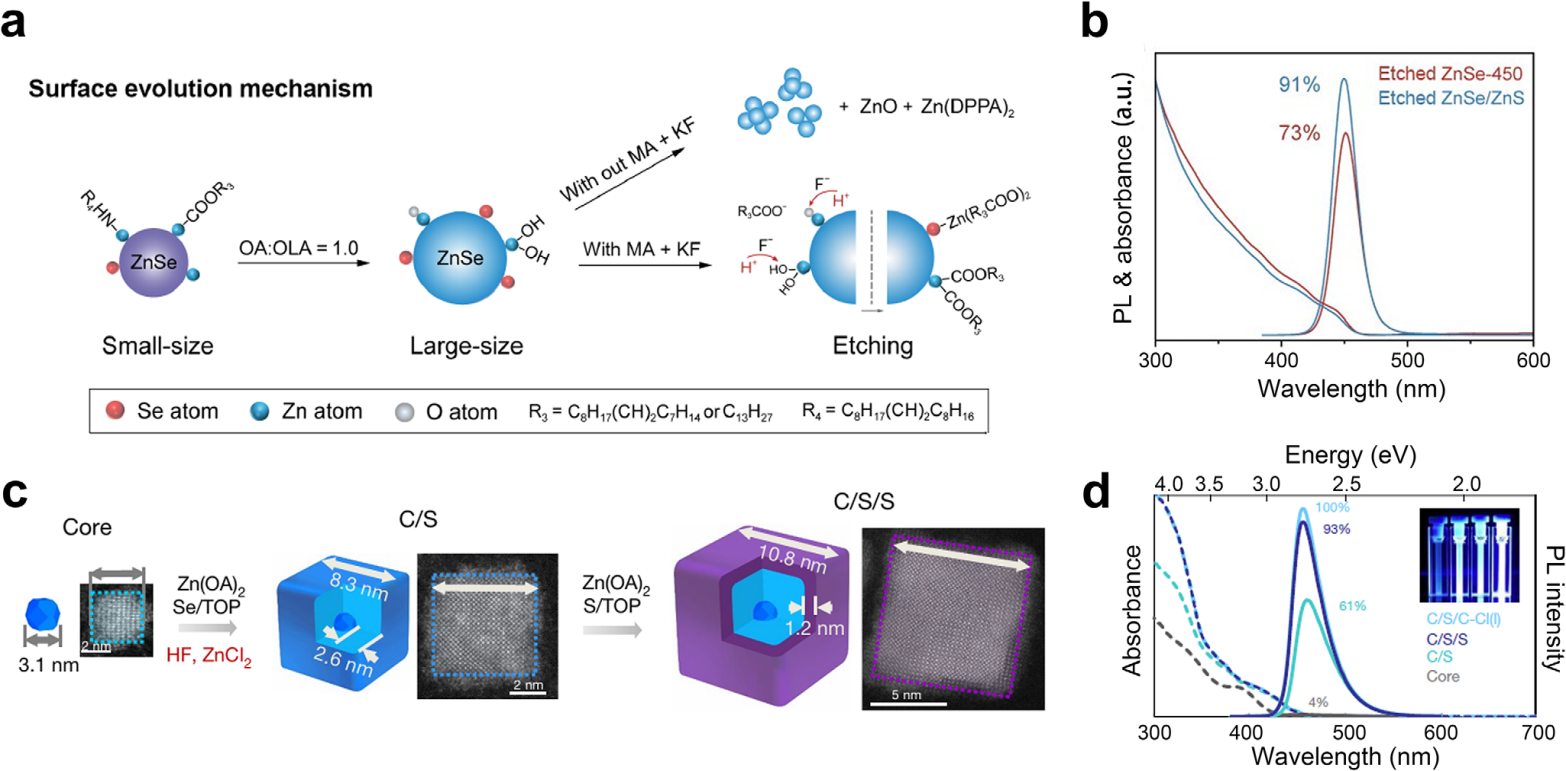
(a) Schematic illustration of surface evolution mechanism at different stages. (b) PL and UV–vis absorption spectra with corresponding PLQYs of etched ZnSe‐450 using KF and MA in combination, and the etched ZnSe/ZnS QDs. Reproduced with permission from ref. [63]. Copyright 2024 Tsinghua University Press. (c) Schematic illustrations of the synthesis of ZnTeSe (core), ZnTeSe/ZnSe (C/S) and ZnTeSe/ZnSe/ZnS (C/S/S) QDs. (d) Absorption and photoluminescence (PL) spectra of the QDs. Inset, photograph taken under illumination at 365 nm; from left to right: core, C/S, C/S/S and C/S/S‐Cl(l)). Reproduced with permission from ref. [49]. Copyright 2020 Springer Nature.

The Zhong group investigated the side reactions that occur during the synthesis of large‐size ZnSe NCs [63]. They observed the turbidity of the colloidal solution and the decrease in PL of the ZnSe cores, which are associated with the side reactions between oleic acid (OA) and oleylamine (OLA). These negative effects can be mitigated by employing a modified etching strategy that combines potassium fluoride (KF) and myristic acid (MA), leading to the formation of large‐size ZnSe/ZnS core/shell QDs with a maximum photoluminescence quantum yield (PLQY) exceeding 90% (Figure 5a,b).

Subsequently, Kim et al. successfully enhanced the PLQY of QDs by incorporating HF and ZnCl_2_ additives to mitigate delamination within the ZnSeTe crystal structure [49]. (Figure 5c,d) Following shell coating, the obtained ZnSeTe/ZnSe/ZnS QDs exhibited a PL peak at 457 nm with a PLQY approaching 100%. (Figure 2d) Herein, the HF separated the ligands via protonation and effectively removed the surface oxidation states through etching. Meanwhile, ZnCl_2_ was employed to regulate the growth direction of the initial ZnSe shell.

### 4.4 Shell Passivation

The epitaxial growth of large bandgap semiconductor materials on the QDs, forming core/shell structure, is a common and effective strategy to passivate the surface sites and isolate from the outside. According to the difference in energy level structure between the core and shell materials, the core/shell structure can be divided into three types, type‐I, type‐II, and quasi‐type‐II.

**FIGURE 6:**
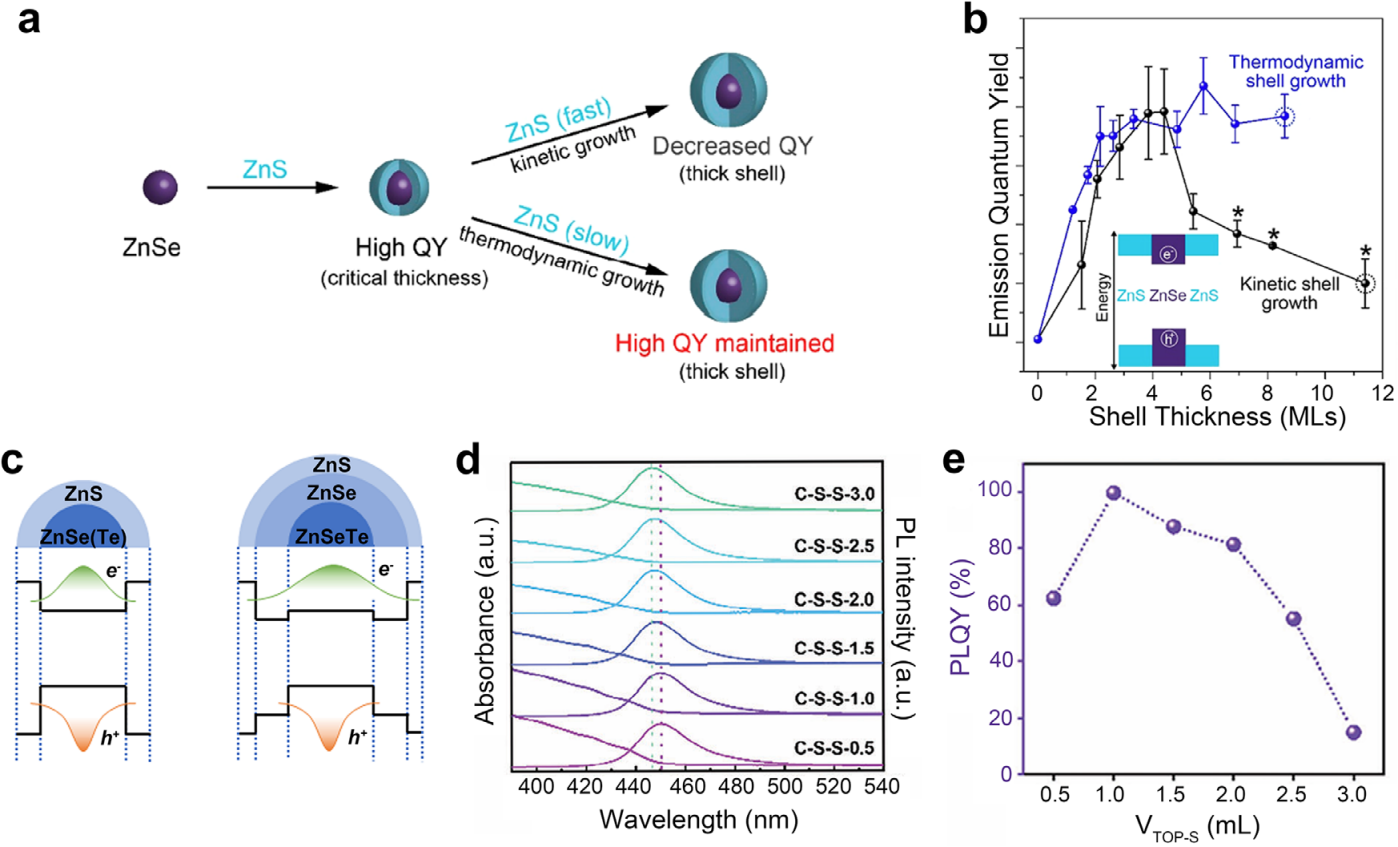
(a) Schematic illustration of the controlled shell growth of ZnS on a spherical ZnSe QD. Stage I, initial ZnS shell growth produces ZnSe/ZnS QDs with high QY when below a critical thickness. Stage II, ZnSe QDs with thick ZnS shells grown in a kinetic regime display decreased QY versus maintained high QY for shell growth in a thermodynamic regime. The thermodynamic growth is realized by using Zn precursor with low reactivity. (b) The PLQY curves of ZnSe/ZnS QDs with the increase of shell thickness for thermodynamic shell growth and kinetic shell growth. Inset is the energy level schematic illustration of ZnSe/ZnS QDs. Reproduced with permission from ref. [64] under CC BY. Copyright 2020 American Chemical Society. (c) Schematic diagrams of the energy band structures and the wave functions of electrons and holes for ZnSe(Te)/ZnS (left) and ZnSeTe/ZnSe/ZnS (right) QDs. (d) Absorption and PL spectra of the ZnSeTe/ZnSe/ZnS QDs with different ZnS shell thickness. (e) The variation of absolute PLQY of the C‐S‐S QDs with increasing TOP‐S injection amount. Reproduced with permission from ref. [68]. Copyright 2024 John Wiley and Sons.

Coating ZnS with a large bandgap can form a type‐I core/shell structure on the surface of ZnSe(Te) QDs. The electrons and holes are confined within the core materials, which decreases the carriers escaping outward and increases the radiative recombination, thereby effectively increasing the PLQY. Controlling the shell thickness is crucial for the passivation of QDs. If the shell is too thin, the surface passivation of the core is insufficient, leading to poor optical stability and facilitating electric field‐induced exciton dissociation. In contrast, an excessively thick shell introduces lattice strain due to lattice mismatch, which promotes the formation of defect states at the core/shell interface and reduces carrier injection efficiency. Meanwhile the shell growth rate also influences the structure and optical properties of QDs. Banin et al. tune the precursor reactivity that modifies the growth mode of ZnS shells on ZnSe cores transforming from kinetic (fast) to thermodynamic (slow) growth regimes [64]. (Figure 6a) Under the thermal growth regime, it effectively avoids the traps at the core–shell interface that are unfavorable for emission properties, leading to an enhanced PLQY and reduced on‐off blinking of ZnSe/ZnS QDs. (Figure 6b).

The initial approach, which involved coating the ZnSeTe core solely with wide bandgap ZnS, proved to be suboptimal. (Figure 6c left) The significant lattice mismatch between core and shell promotes the formation of interfacial defects at the core–shell boundary, which serve as exciton trapping sites. These non‐radiative recombination centers reduce radiative recombination efficiency, thereby decreasing the overall PLQY. To address this limitation, researchers have proposed a heterojunction structure utilizing a ZnSeTe/ZnSe quasi‐type‐II band alignment. With subsequent passivation through a ZnS shell, this design achieves a PLQY approaching 100%, representing the most effective and rationally designed shell engineering strategy reported to date [49]. This multi‐layered shell architecture significantly mitigates lattice mismatch between the core and shell. This mechanism effectively reduces interfacial strain across the core–shell and shell–shell boundaries, thereby improving PLQY. Moreover, in heterojunction‐structured QDs, the electron wave function tends to delocalize within the ZnSe shell, whereas the hole wave function remains spatially confined to the core region, which will leading a redshift of PL spectrum of QDs [65] (Figure 6c right).

Therefore, precise control over the thickness of both the ZnSe and the ZnS shell is crucial for enhancing carrier recombination efficiency and optimizing PL performance. Lee et al. precisely controlled the growth durations of ZnSe and ZnS shells, enabling the synthesis of QDs with tunable emission wavelengths ranging from 451 to 463 nm and FWHM between 18 and 38 nm [66]. On this basis, Bi et al. demonstrated that precise control over the ZnSe shell thickness effectively suppresses exciton‐LO phonon coupling and mitigates defect‐related carrier trapping in QDs, thereby achieving a narrow FWHM and high PLQY [67]. Furthermore, Cheng et al. synthesized ZnSe and ZnS shells on ZnSeTe QDs using a stepwise injection approach [68]. They effectively mitigated lattice strain, minimized surface and interfacial defects, and achieved a uniform ZnSe‐ZnS double‐shell structure with tunable thickness. (Figure 6d) The resulting QDs exhibited a PL peak at 448 nm, a narrow FWHM of 23 nm, and a PLQY approaching 100% (Figure 6e).

However, there is still a ∼5% lattice mismatch between ZnSe and ZnS, which will lead to interface defects and hinder the carriers injection. Therefore, the inner alloy layer is imported to form gradient energy levels, constructing ZnSe/ZnSeS/ZnS and ZnSeTe/ZnSe/ZnSeS/ZnS gradient‐shell QDs. Jung et al. synthesized ZnSe/ZnSeS/ZnS gradient QDs, which exhibited an enhanced PLQY from 63.7% for ZnSe/ZnS QDs to 77.2% [69]. (Figure 6e) Meanwhile, ZnSeTe/ZnSe/ZnSeS/ZnS QDs also have been attempted to construct [54, 70].

### 4.5 Ligand Engineering

Besides the shell layers, the surface ligands of QDs also play a critical role in determining their environmental stability (such as resistance to moisture, oxygen, thermal stress, and ultraviolet radiation) as well as influencing surface defect states and PL properties. This approach can passivate the surface defects of QDs to reduce defect trapping, eliminate excessive insulating ligands to promote efficient carrier transport, and has already been identified as an effective method for enhancing the efficiency and stability of QDs.

**FIGURE 7:**
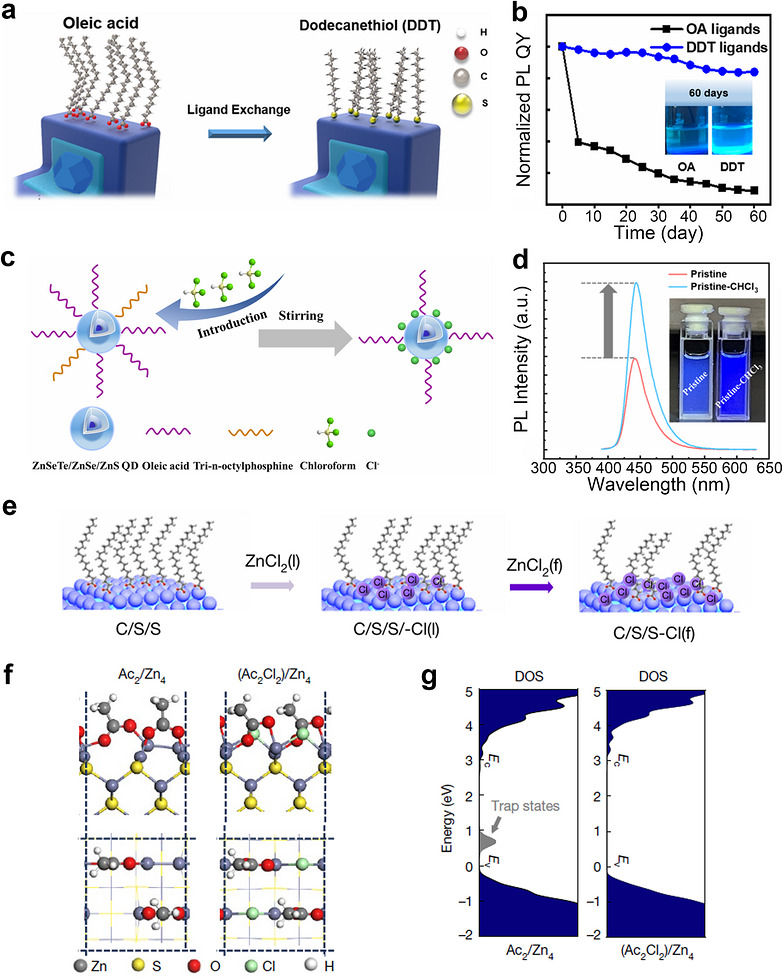
(a) Representative schematic illustration of the ligand exchange from oleic acid (OA) to alkanethiol ligands. (b) PL QY stability data of ZnCl_2_‐C/S/S QDs and DDT‐treated QDs under ambient conditions (insets: the comparison of the luminance between two samples under UV light (365 nm)). Reproduced with permission from ref. [71]. Copyright 2022 American Chemical Society. (c) Schematic illustration of chloroform post‐treatment ZnSeTe/ZnSe/ZnS QDs. (d) Comparisons of photoluminescence spectra along with a fluorescent image of QD solution under UV lamp (inset). Reproduced with permission from ref. [72]. Copyright 2024 American Chemical Society. (e) Schematic drawing of the ligand exchange with ZnCl_2_ in the liquid phase (ZnCl_2_ (l)) and further exchange through film‐washing treatment (ZnCl_2_ (f)). (f) Relaxed configurations of ZnS (100) surfaces with ligands for the under‐passivated (Ac2/Zn4) and the fully passivated (Ac_2_Cl_2_/Zn_4_) states. (g) Calculated density of states (DOS) for Ac_2_/Zn_4_ and (Ac_2_Cl_2_)/Zn_4_ surfaces. *E_v_*, valence band maximum; *E_c_*, conduction band minimum. Reproduced with permission from ref. [49]. Copyright 2020 Springer Nature.

During the synthesis of QDs, long‐chain carboxylic acid ligands are commonly employed to modulate the reaction process and passivate surface metal sites. However, the effectiveness of these organic ligands in surface passivation is limited due to steric hindrance and their relatively weak binding affinity toward QDs, which may adversely affect colloidal and structural stability. Therefore, the selection of ligands with strong binding affinity is crucial for enhancing the overall stability of QDs. Cho et al. employed a ligand exchange strategy to substitute the OA ligands on the QDs surface with dodecanethiol (DDT) ligands, which possess stronger binding affinity, thereby significantly enhancing the stability of the QDs [71]. (Figure 7a) The PLQY remained above 80% of the initial value even after 60 days of exposure to air (Figure 7b).

Despite the strong binding capability of thiol ligands, steric hindrance effects can result in incomplete surface passivation of QDs. Therefore, the design of high‐density ligand coatings may significantly enhance colloidal stability. Halogen ligands are regarded as highly effective passivating agents owing to their compact molecular configuration and strong antioxidant characteristics. Zheng et al. developed a chloroform‐based post‐treatment strategy, in which Cl^−^ are generated through the reaction between chloroform and TOP ligands. These Cl^−^ effectively passivate surface defects on QDs, resulting in a significant enhancement of PLQY from 39.6% to 92.0% [72]. (Figure 7c,d) Kim et al. repeatedly treated the surface of QDs with ZnCl_2_, resulting in QDs exhibiting a high PLQY approaching 100% [49]. (Figure 7e) Density of states (DOS) calculations revealed prominent defect states in untreated QDs, as illustrated in Figure 7f,g. The incorporation of Cl^−^ effectively passivated these defect states, leading to a significant enhancement in the radiative recombination efficiency of the QDs.

**FIGURE 8:**
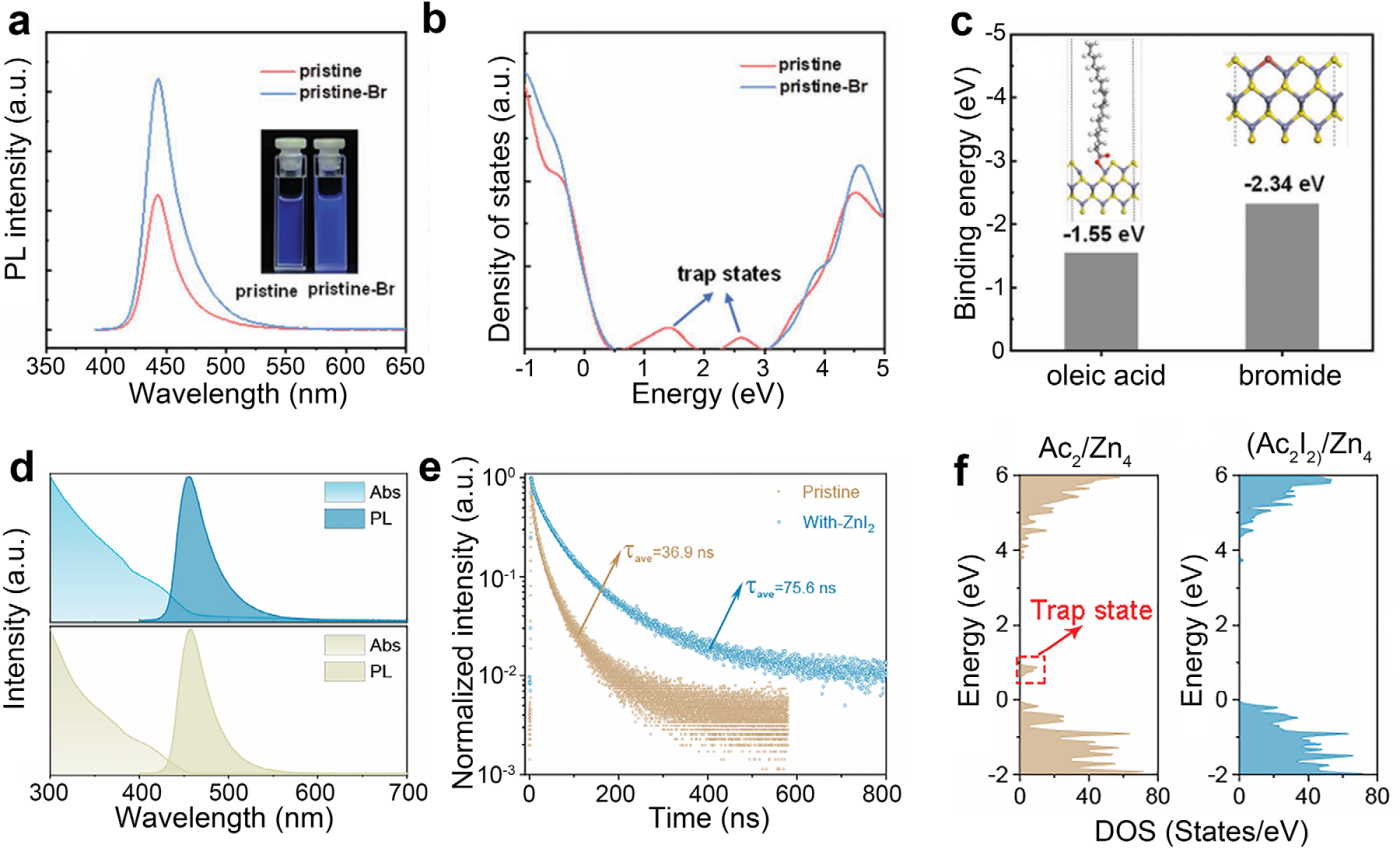
The ligand engineering of ZnSeTe QDs: (a) Steady‐state photoluminescence (inset: the fluorescent pictures of QD solution under UV‐lamp) of pristine and pristine‐Br QDs. (b) Density of states (DOS) of ZnS shell on pristine and pristine‐Br QD surface. (c) Density functional theory (DFT) simulations of the binding energy of oleic acid and bromide with Zn of the QD surface. Reproduced with permission from ref. [73]. Copyright 2022 John Wiley and Sons. (d) Absorption and PL spectra, and (e) TRPL of the pristine and with‐ZnI_2_ QDs. (f) Calculated density of states (DOS) for Ac_2_/Zn_4_ and (Ac_2_I_2_)/Zn_4_ surfaces. Reproduced with permission from ref. [74]. Copyright 2024 John Wiley and Sons.

In another study, Zheng et al. replace the OA ligands with Br^−^ on the QDs surface, thereby effectively reducing surface defect states and enhancing the PLQY from 39.7% to 86.2% [73]. (Figure 8a) Density functional theory (DFT) calculations further confirm that the trap states are effectively passivated by Br^−^, and the binding energy between Zn and Br on the QDs surface is significantly higher than that between Zn and OA. (Figure 8b,c) Guan et al. employed ZnI_2_ to passivate the QD, thereby effectively reducing surface defects and enhancing the PLQY from 45% to 63% [74]. (Figure 8d) Theoretical calculations further revealed that the binding energy between Zn dangling bonds and I^−^ on the QDs surface is notably greater than that between Zn and OA. (Figure 8e,f) These findings suggest that halogen‐based ligands, owing to their strong affinity for surface Zn dangling bonds, serve as highly efficient passivating agents for QD surface engineering.

## 5 Blue ZnSe(Te) QLEDs Applications and Optimization

**FIGURE 9:**
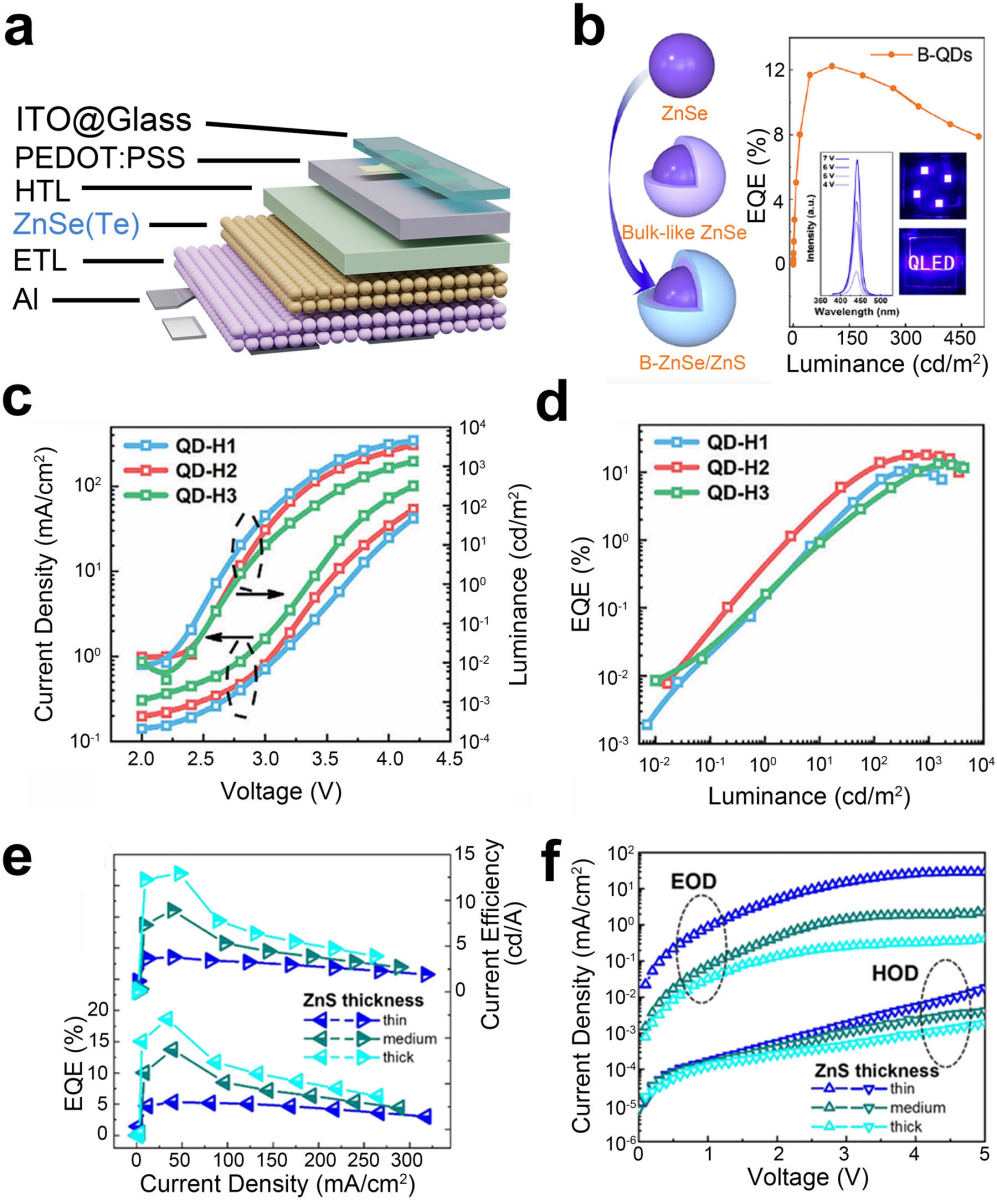
(a) The schematic device structures of QLEDs based on ZnSe(Te) QDs. (b) The structural evolution diagram of bulk‐like ZnSe/ZnS QDs and the corresponding device electroluminescent performance. Reproduced with permission from ref. [53]. Copyright 2021 American Chemical Society. (c) Current density‐voltage, luminance‐voltage, and (d) EQE‐luminance relations for QLEDs based on QD‐H1, QD‐H2, and QD‐H3, respectively. Reproduced with permission from ref. [67]. Copyright 2023 John Wiley and Sons. (e) current efficiency and EQE‐current density relations of blue devices as a function of ZnS outer shell thickness. (f) Current density‐voltage characteristics of EODs and HODs as a function of ZnS outer shell thickness. Reproduced with permission from ref. [66]. Copyright 2022 Elsevier.

Based on the high‐performance ZnSe(Te) QDs, the evaluation of electroluminescence (EL) properties, which can be collected by the light‐emitting diodes (LEDs), is an important step toward the display applications. Compared with the PL from the exciton recombination, the EL emission comes from the injection and recombination of the carriers. The typical EL device structure is a multilayered structure. Typically, the device structures are employed as ITO/PEDOT:PSS/hole‐transport layer (HTL)/emitting layer (EML)/electron‐transport layer (ETL)/Al (Figure 9a).

Xiang et al. were among the first to report the synthesis of cadmium‐free ZnSe/ZnS QDs and the fabrication of EL devices based on these materials [75]. ZnSe/ZnS QDs were synthesized via a hot‐injection method, resulting in a peak emission wavelength of 420 nm, a FWHM of 16 nm, and a PLQY of 40%. Subsequently, the LEDs with a structure of ITO/PEDOT:PSS/PVK/QDs/ZnO/Al were fabricated, demonstrating blue‐violet EL with a maximum EQE of 0.65%. The Yang Group has successfully developed the first blue‐emitting ZnSeTe QLED device with a structure of ITO/PEDOT:PSS/PVK/QDs/ZnMgO/Al, achieving stable EL at 445 nm with a maximum luminescence of 1195 cd/m^2^ and a maximum EQE of 4.2% [40]. At the time, these performance metrics represented the highest values reported for Cd‐free QDs. Research based on ZnSe(Te) systems LEDs was scarce, and the EQEs of almost all reports were below 10% until 2020. Moreover, the EL peaks always locate in the violet region (420–440 nm) due to the inherent wide bandgap of ZnSe QDs. The poor properties mainly come from two aspects: the low PLQY of QDs themselves and imbalanced carriers injection of QLEDs device. In order to improve the EL properties of blue‐emitting devices, researchers have developed several strategies to enhance the performance.

### 5.1 EML Optimization

#### 5.1.1 Shell Structure

For QDs materials, researchers initially concentrated on optimizing the shell structure. As previously discussed, ZnSe(Te) QDs can significantly enhance PL performance and reduce FWHM through precisely controlling of the shell structure and thickness. For ZnSe QDs, the gradient core–shell structure ZnSe/ZnSeS/ZnS was constructed by Jung et al., enabling the realization of pure‐blue‐emitting ZnSe‐based devices with an emission peak exceeding 450 nm [69]. The QLEDs exhibited an EQE of 5.32% and a high luminescence of 3754 cd m^−2^. Although the EL peak of ZnSe QDs can be tuned to the blue‐emitting region, the FWHM has broadened significantly, which affects the color purity. Shen et al. adopted a simple epitaxial growth strategy, growing a ZnSe layer on the ZnSe core, obtaining a bulk‐like size ZnSe cores, which enables the emission to locate in the blue region with a narrow emission width close to its intrinsic peak width. Utilizing this ZnSe/ZnS QDs, the fabricated QLED demonstrates a peak EL wavelength of 445 nm, a FWHM of less than 12 nm, a peak EQE of 12.2%, and a maximum luminescence of 1055 cd/m^2^ [53]. (Figure 9b) It demonstrates outstanding operational stability, as evidenced by a half‐lifetime (T_50_) of 237 h. Furthermore, to meet the requirements for blue LEDs in display applications, Zhong group developed a reaction activity‐controlled epitaxial growth strategy to synthesize large‐diameter ZnSe/ZnS NCs with high PLQY [52]. Building upon this approach, they successfully developed a pure blue LED at 456 nm with a narrow FWHM of 22 nm, which exhibits a maximum EQE of 4.2% and a peak luminance of 1,223 cd/m^2^ [63].

The shell thickness of the multilayer shell structure will affect QDs EL properties, including the luminescence properties and carrier injection. Bi et al. fabricated LED devices based on ZnSeTe QDs with varying ZnSe shell thicknesses, categorized as thin, medium, and thick [67]. (Referring respectively to QD‐H1, QD‐H2, and QD‐H3 in Figure 9c,d). The QLED device incorporating a medium‐thickness ZnSe shell exhibited the highest EQE of 18%. A relatively thick ZnSe shell was observed to suppress the Förster resonance energy transfer (FRET) process, while simultaneously promoting balanced carrier transport through the reduction of energy barrier levels. In addition to the inner ZnSe shell, the outer ZnS shell also plays a significant role in modulating the EL performance. Lee et al. investigated the performance of LED devices based on ZnSeTe QDs with varying ZnS shell thicknesses [66]. As the ZnS shell thickness increased, the peak luminescence of the devices gradually improved, with the highest EQE achieved in the sample with thickest ZnS shell, reaching 18.6%. (Figure 9e) Through the analysis of devices with electron‐only and hole‐only, it was found that a thicker ZnS shell suppresses the electron injection rate more significantly than the hole injection rate. (Figure 9f) This effect contributes to a more balanced carrier injection, thereby improving device efficiency.

#### 5.1.2 Ligands Engineering

On the other hand, ligand engineering of QDs also plays a crucial role in enhancing LED performance. During the synthesis of QDs, long‐chain organic carboxylic acids are commonly employed as ligands to control the reaction process and stabilize surface metal sites. However, the steric hindrance induced by these bulky organic ligands increases the interparticle distance between adjacent QDs, resulting in the formation of an insulating barrier that impedes charge carrier injection. Wang et al. developed a novel “low temperature injection and high temperature growth” method, fabricating blue‐violet ZnSe/ZnS QDs with a high PLQY (83%) and excellent stability. By using octanethiol (OT) instead of S‐ODE as both the sulfur source and ligand and leveraging the strong bonding between OT and the surface of QDs, the stability of QDs under UV irradiation and during the repeated purification process was significantly enhanced [50]. The short‐chain ligand also facilitated charge injection, thereby increasing the EQE of the device from 2.6% for QD based on S‐ODE to 7.83% for QD based on OT, with an emission peak of 429 nm, which was the highest reported value at that time.

**FIGURE 10:**
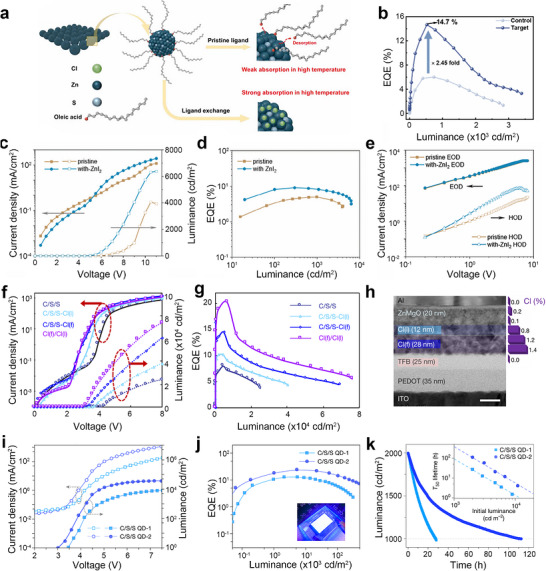
The EL performance of ZnSeTe QLED: (a) Schematic illustration of solid‐phase ion‐ligand exchange. (b) Luminance dependence of the EQE. Reproduced with permission from ref. [76]. Copyright 2024 American Chemical Society. (c) Current density‐luminance‐voltage (*J*–*L*–*V*) curves and (d) luminance‐external quantum efficiency (L‐EQE) characteristics. (e) The current density curve corresponding to the single carrier device. Reproduced with permission from ref. [74]. Copyright 2024 John Wiley and Sons. (f) Voltage‐dependent current density (left axis) and luminance (right axis). (g) Luminance dependence of the EQE. (h) Cross‐sectional TEM image (scale bar, 30 nm) of the QD‐LED with double EML. Reproduced with permission from ref. [49]. Copyright 2020 Springer Nature. (i) *J*–*V*–*L* and (j) EQE–*J* curves of QLEDs. (k) Operational lifetime for QLEDs with C/S/S QD‐1 and QD‐2. Inset: corresponding T_50_ lifetime at different initial luminance. Reproduced with permission from ref. [60]. Copyright 2025 Springer Nature.

As previously discussed, inorganic halide ions demonstrate strong binding affinity with Zn dangling bonds on the QD surface, enabling efficient and dense passivation of surface defect states, thus enhancing PL performance. Zheng et al. utilized a reaction between chloroform and TOP ligands to generate Cl^−^, which passivated the QDs surface [72]. This treatment elevated the valence band maximum of QDs from −6.59 to −6.29 eV, thereby significantly improving the hole injection efficiency. As a result, the performance of fabricated QLED was enhanced, with the EQE increasing from 0.62% to 2.25%. Furthermore, Fei et al. developed a solid‐phase ion‐ligand exchange strategy that employs ZnCl_2_ as a short‐chain conducting ligands to dispose the QDs film, thereby improving the thermal stability and carrier injection capacity of the ZnSeTe QD films [76]. (Figure 10a) QLEDs fabricated through this strategy exhibit more balanced charge carrier injection, resulting in outstanding performance in pure blue (460 nm) QLEDs, with a peak EQE of 14.7% and an extended T_50_ of 52.7 h (@100 cd/m^2^), representing 2.45‐ and 7.1‐fold improvements over the control device, respectively (Figure 10b).

As reported by Zheng et al., Br^−^ were employed to passivate surface defect states of QDs, thereby significantly enhancing their PLQY [73]. During the spin‐coating process of QDs onto the hole transport layer, it was observed that the QD films without Br treatment exhibited numerous cracks, whereas the Br‐treated QD films demonstrated a smooth surface and uniform surface morphology. The surface roughness of the QD films decreased from 13.8 to 5.1 nm, which can be attributed to reduced interparticle spacing and a more compact arrangement of the QDs. Consequently, the performance of the QLED improved by approximately 7‐fold, with the EQE increasing from 0.74% to 5.46%. Furthermore, Guan et al. employed I^−^ to modify the surface of QDs [74]. The valence band maximum of the QDs shifted from −6.26 eV prior to treatment to −6.14 eV afterward, effectively reducing the hole injection barrier. Characterization based on single carrier devices demonstrated that the electron transport rate remained largely unchanged after I^−^ treatment, whereas the hole transport rate was significantly improved. This surface modification also enhanced device conductivity, leading to an approximately two‐fold increase in the performance of the fabricated QELD. A maximum luminescence with 6,370 cd/m^2^ was achieved, along with a maximum EQE of 9.1% (Figure 10c–e).

Building upon QDs exhibiting near 100% PLQY, Kim et al. successfully achieved efficient passivation of QDs via a Cl^−^‐based dual‐phase ligand exchange process involving both liquid and solid phases [49]. This approach conferred exceptional thermal stability and efficient charge transport characteristics to the QDs. Building on this foundation, the researchers developed a dual QDs layer architecture with a gradient distribution of Cl^−^, which significantly enhanced hole transport and resulted in near‐theoretical EQE. The resulting device achieved a high EQE of 20.2%, a maximum luminescence of 88 900 cd/m^2^, and an extended operational lifetime with T_50_ reaching 15 850 h (@100 cd/m^2^). (Figure 10f–h) This study underscores the significant potential of ZnSeTe quantum dots for applications in blue light emission.

Besides these two main strategies, the Yang group innovatively introduced highly electronegative S^2^^−^ into the ZnSeTe core, which effectively suppressed hole localization at Te atoms and narrowed the FWHM of the QDs [60]. Moreover, the congeneric S enhances the configurational entropy of the QDs and eliminates the stacking faults as well as oxygen defects, resulting in improved structural stability and a reduced non‐radiative carrier density. This modification LED to significant advancements in QLED devices based on ZnSeTeS QDs, achieving an EQE of 24.7% at a wavelength of 460 nm, a FWHM of 17 nm, and a T_50_ operational lifetime of nearly 30 000 h (@100 cd/m^2^), which represents the highest EL properties reported to date for Cd‐free blue QLEDs (Figure 10i–k).

### 5.2 Hole Transport Layer

**FIGURE 11:**
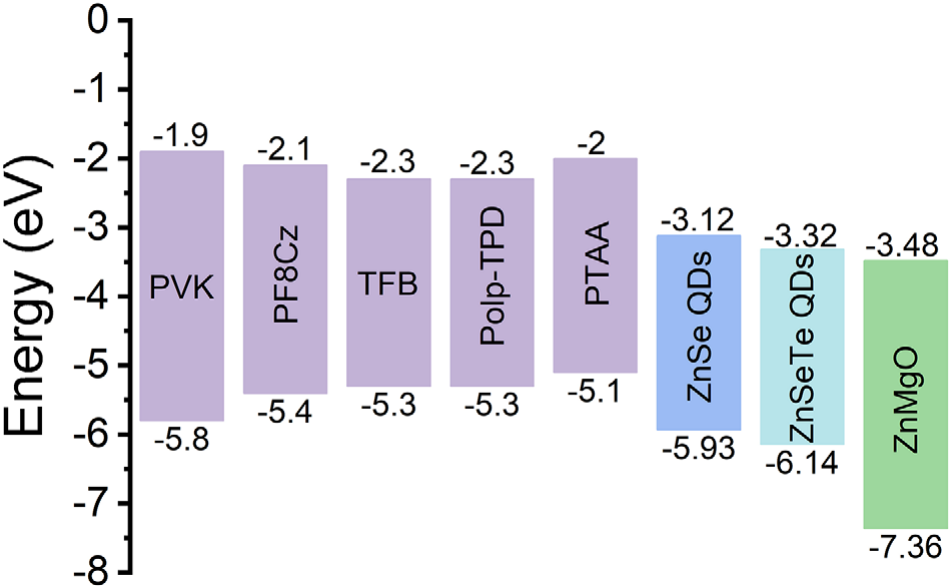
Energy levels of the commonly used hole transport layer materials (purple), electron transport layer materials (green), and ZnSe(Te) QDs. The full names of some carrier transport layer materials are summarized here: poly(9‐vinlycarbazole) (PVK), poly((9,9‐dioctylfluorenyl ‐2,7‐diyl)‐alt‐(9‐(2‐ethylhexyl)‐carbazole‐3,6‐diyl)) (PF8Cz), poly[(9,9‐dioctylfluorenyl‐2,7‐diyl)‐co‐(4,4’‐(N‐(4‐sec‐butylphenyl)) diphenyl‐amine) (TFB), poly[N,N’‐bis(4‐butylphenyl)‐N,N’‐bis(phenyl)benzidine] (poly‐TPD), [bis(4‐phenyl)(2,4,6‐trimethylphenyl)amine] (PTAA).

In addition to the optimization of QDs materials, optimizing device structure is equally crucial for enhancing the performance of QLEDs. The primary objective is to achieve balanced carrier injection and mitigate efficiency losses associated with carrier over‐injection. In a conventional QLED structure, organic polymer molecules are typically employed as the HTL, whereas ZnO nanoparticles serve as the ETL, collectively forming a sandwich structure. However, a high potential barrier typically exists at the interface between the HTL and the EML, while the electron injection barrier is comparatively low. This discrepancy results in an imbalance in carrier injection dynamics. Therefore, enhancing the hole mobility is the primary target. According to Figure 11, the different HTL exhibit different HOMO energy level and different hole mobility. Thus, the selection of HTL to balance the hole injection barrier and holes mobility is crucial for the carrier balance to enhance the EL properties.

Among the available materials, TFB and PVK are the most widely employed materials for HTL. TFB demonstrates a relatively high hole mobility of 1 × 10^−^^22^ cm^2^ V^−^^1^ s^−^^1^, while its HOMO energy level is comparatively shallow (−5.3 eV). In contrast, PVK exhibits a lower hole mobility of 2.5×10^−^^6^ cm^2^ V^−^^1^ s^−^^1^, but possesses a deeper HOMO energy level (−5.8 eV), which enables more favorable alignment with the valence band maximum of QDs [4]. Bao et al. incorporated 10% TFB into PVK, effectively combining the high hole transport capability of TFB with the deeper HOMO energy level of PVK to fabricate a ZnSeTe QLED exhibiting an EQE of 0.33%, surpassing the performance of devices based on pristine TFB (0.12%) or PVK (0.18%) [77]. The low efficiency was mainly attributed to the low PLQY of 30% for QDs. Furthermore, Shen group developed ZnSe/ZnS QDs with high PLQY and systematically optimized the HTL through comprehensive material screening [71]. By integrating TFB with PVK, they effectively balanced device performance in terms of turn‐on voltage, luminance, and EQE. Consequently, they successfully fabricated a blue‐violet LED exhibiting an EQE of 7.39% and a maximum luminance of 2856 cd/m^2^. Further improvements in QDs performance have resulted in an enhancement of the EQE to 7.83% [72].

### 5.3 Electron Transport Layer

Compare with enhance the hole mobility, reducing the electron mobility is a more efficient strategy. Single ZnO nanoparticles commonly exhibit too fast electron mobility [78], which is fast than hole mobility. To reduce the electron mobility, researchers commonly employ metal ion doping ZnO, such as Mg, Li, and Al to effectively reduce the electron mobility [79]. Mg^2+^ doped ZnO nanoparticle (ZnMgO) have been proven to slow down the electron mobility of ZnO and enhance device performance [80]. As the doping amount of Mg increases, the mobility gradually decreases.

**FIGURE 12:**
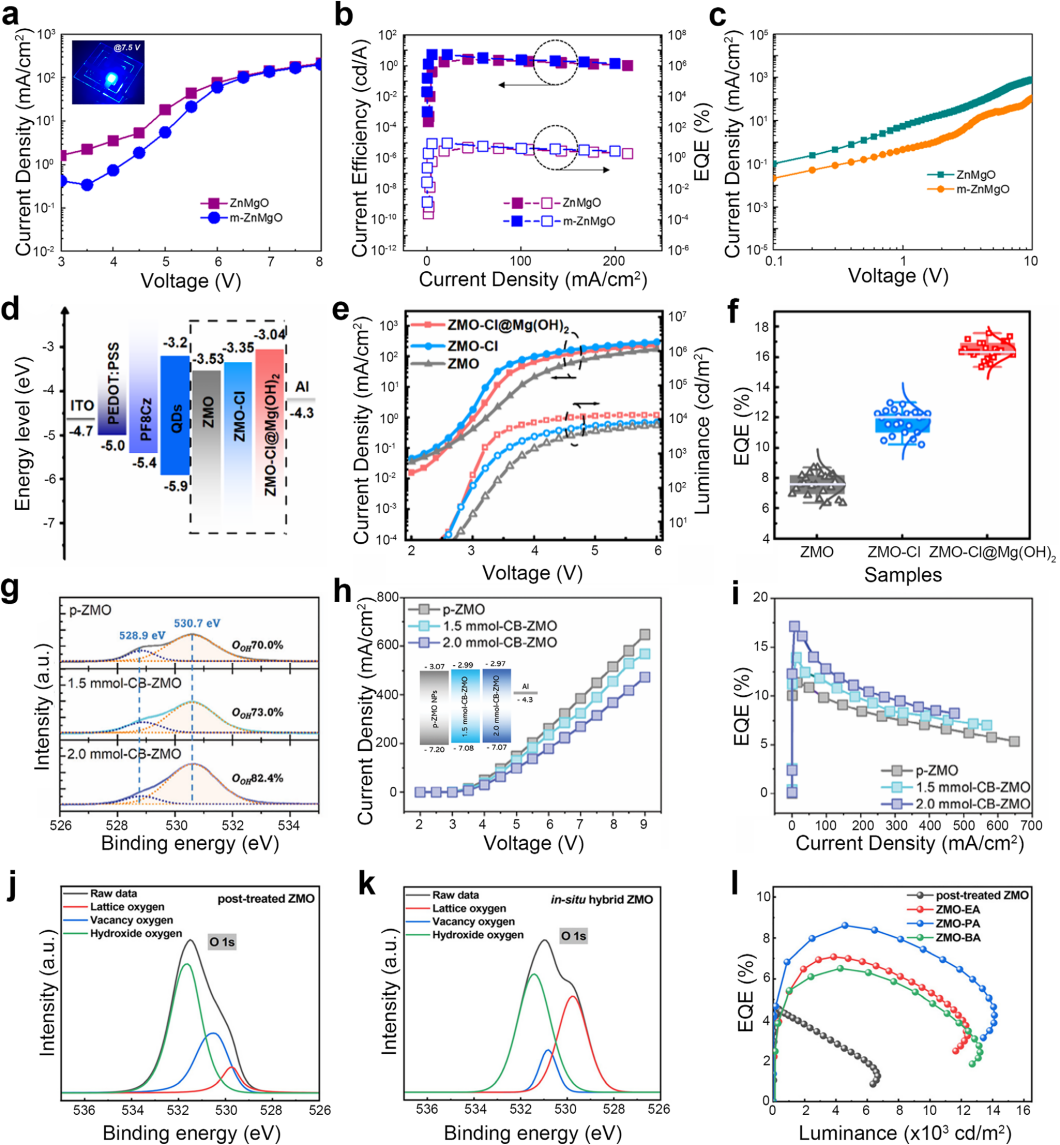
The ETL optimization of ZnSeTe QLED: Variation of (a) current density with applied voltage (inset, EL image collected at 7.5 V), and (b) current efficiency−EQE as a function of current density for pristine ZnMgO NP ETL‐based vs m‐ZnMgO NP ETL‐based QLEDs. (c) Comparison of current density−voltage traces of pristine ZnMgO and m‐ZnMgO NP ETL‐based electron‐only devices consisting of an ITO/ETL/QD/ETL/Al structure. Reproduced with permission from ref. [81]. Copyright 2020 American Chemical Society. d) Energy level diagram of the devices. (e) Current density–voltage–luminance (*J*−*V*−*L*) characteristics curves of QLEDs. (f) Efficiency reproducibility of devices based on ZMO, ZMO‐Cl and ZMO‐Cl@Mg(OH)_2_ ETLs. Reproduced with permission from ref. [82]. Copyright 2025 American Chemical Society. (g) high‐resolution O 1s XPS scans of p‐ZMO and bicarbonate‐functionalized ZMO NPs with different CB amounts. Variations of (h) luminance‐voltage (inserts, schematic of ETL‐dependent energy band levels, showing gradual energetic upshift with increasing CB amount) and (i) EQE−current density of blue QLEDs with different ETLs. Reproduced with permission from ref. [83]. Copyright 2024 John Wiley and Sons. XPS spectra of O 1s for the (j) post‐treated ZMO and (k) in situ hybrid ZMO films. (i) EQE–luminance performance comparison of QLEDs using the post‐treated ZMO, ZMO‐EA, ZMO‐PA and ZMO‐BA as ETLs. Reproduced with permission from ref. [84]. Copyright 2024 The Royal Society of Chemistry.

Han et al. developed an additional Mg reaction modification strategy on the surface of ZnMgO nanoparticles, resulting in the formation of a Mg(OH)_2_ protective layer [81]. This layer significantly reduced electron mobility and mitigated fluorescence quenching in QDs. By employing the modified ZnMgO@Mg(OH)_2_ as ETL in QLED, the maximum luminescence increased from 2305 to 2904 cd/m^2^, and the maximum EQE improved significantly from 4.7% to 9.5%. The external Mg(OH)_2_ layer functioned as an insulating barrier, effectively suppressing electron injection and thereby enhancing device performance (Figure 12a–c). Building upon this foundation, Sun et al. further introduced Cl^−^ to passivate oxygen vacancies on the surface of ZnMgO nanoparticles, followed by the application of a Mg(OH)_2_ protective layer [82]. The QDs above ZnMgO‐Cl@Mg(OH)_2_ film exhibited the highest PL intensity and the lowest quenching efficiency. The incorporation of Cl^−^ effectively passivated surface defect sites of ZnMgO nanoparticles, thereby reducing fluorescence quenching at the QDs/ETL interface. Subsequent shell coating effectively suppressed Cl^−^ migration, contributing to a comprehensive improvement in device performance. The optimized QLED achieved a maximum EQE of 17.5% at 6.0 V and a maximum luminescence of 13 670 cd/m^2^, representing a two‐fold improvement over the original ZnMgO‐based device. Additionally, the operational stability was enhanced by 4.6 times, with a T_50_ lifetime of 5.1 h (@1000 cd/m^2^) (Figure 12d–f).

In addition to providing shell protection, Yoon et al. functionalized the ZnMgO surface with bicarbonate ions [83]. Analytical results indicated that this bicarbonate modification significantly reduced surface trap states in ZnMgO, particularly oxygen vacancies (V_O_), thereby enhancing chemical stability and effectively suppressing interfacial exciton quenching between ELT and EML. Furthermore, the presence of bicarbonate on the surface induced an upward shift in the conduction band minimum of the ETL via the dipole effect. This shift increased the energy barrier at the ETL/Al interface, reducing electron injection efficiency and contributing to more balanced carrier injection. As a result, the EQE of the optimized QLED improved from 11.4% to 17.1%, and the maximum luminescence increased from 31 707 to 39 739 cd/m^2^. Furthermore, the device demonstrated an extended operational stability, achieving a T_50_ lifetime of 42.2 h at 2000 cd/m^2^, which corresponds to an estimated operational duration of approximately 8224 h at 100 cd/m^2^. (Figure 12g–i) Yang group developed an in situ hybridization strategy by incorporating amino alcohol compounds during the synthesis of ZnMgO [84]. This approach effectively suppressed the affinity condensation reaction, reduced the nanoparticles growth rate, and promoted Mg─O bonding formation, thereby enabling the generation of ZnMgO nanoparticles with reduced particle size and fewer structural defects. Consequently, the peak EQE of the fabricated QLEDs improved from 4.5% to 8.6%, the maximum luminance increased from 6668 to 14 125 cd/m^2^, and the T_50_ operational lifetime was extended from 190 to 1301 h at 100 cd/m^2^. (Figure 12j–l) Liang et al. developed a dual passivation strategy by utilizing the P = O containing organic small molecule 2,4,6‐tris(3‐(diphenylphosphoryl)phenyl)‐1,3,5‐triazine (POT_2_T) to simultaneously modify QD film and ZnMgO nanoparticles [85]. This treatment significantly reduced surface defect states in the QDs, leading to an improvement in the PLQY of the QD film. Moreover, the passivation of surface defects on ZnMgO nanoparticles suppressed undesirable interfacial charge transfer and enhanced exciton recombination efficiency. As a result, the fabricated QLED demonstrated a substantial increase in EQE, rising from 10.76% to 18.02%, along with a T_50_ operational lifetime that improved from 143 to 618 h at 100 cd/m^2^. In conclusion, effective passivation of defects in ZMO nanoparticles is crucial to improving the operational stability and performance of QLED devices.

Except ZnMgO as ETL, other ions doping ZnO to reduce the electron mobility also have been attempted. Gao et al. employed ZnSnO as the electron transport layer (ETL) to replace ZnO, thereby reducing the electron transport rate to achieve balanced the carrier injection and minimizing exciton quenching of ZnSe/ZnS QDs, which enhances the probability of exciton recombination [86]. The EQE of the final fabricated ZnSe/ZnS LED increased from 5.1% to 13.6%, and the operational lifetime improved by 21‐folds, reaching 305 h (T_50_ at 100 cd/m^2^). The summarized device structure and performance are shown in Table 1.

**TABLE 1:** Summarized device structure and performance of ZnSe(Te) QDs.

QDs	Device structure	EL Wavelength (nm)	EL FWHM (nm)	V_on_ (V)	EQE (%)	Max Luminance (cd/m^2^)	Lifetime (T_50_@100 cd/m^2^)	Reference
ZnSe/ZnS	ITO/PEDOT:PSS/PVK/QDs/ZnO/Al	420	16	—	0.65	—	—	2012 [75]
ZnSe/ZnS	ITO/ZnO/QDs/CBP/MoO_3_/Al	441	15.2	4	—	1,170	—	2013 [87]
ZnSe/ZnS	ITO/MoO_3_/poly‐TPD/QDs/TPBi/LiF/Al	441.5	17.2	3.9	—	542.1	—	2013 [88]
ZnSe/ZnS	ITO/PEDOT:PSS/TFB:PVK/QDs/ZnO/Al	429	21	6.4	7.39	2,856	—	2015 [89]
ZnSe/ZnS	ITO/PEDOT:PSS/PVK/QDs/ZnO/Al	429	16.6	7	7.83	2,632	—	2015 [50]
ZnSe/ZnSeS/ZnS	ITO/ZnMgO/QDs/TCTA/MoO_3_/Ag	452	22.3	4.01	5.32	3,754	1.27 h	2020 [69]
ZnSe/ZnS	ITO/PEDOT:PSS/PVK/QDs:TPD/ZnMgO/Al	434	16	5	6.88	450	—	2020 [90]
ZnSe/ZnS	ITO/PEDOT:PSS/PVK/QDs/ZnMgO/Al	445	12	4	12.2	1,055	237 h	2021 [53]
ZnSe/ZnS	ITO/PEDOT:PSS/PVK/QDs/ZnSnO/Al	443	12	4.1	13.6	1,031	305 h	2022 [86]
ZnSe/ZnS	ITO/PEDOT:PSS/TFB/QDs/ZnMgO/Al	456	22	2.7	4.2	1,223	—	2024 [63]
ZnSeTe/ZnSe/ZnS	ITO/PEDOT:PSS/PVK/QDs/ZnMgO/Al	445	32	4	4.2	1,195	5 min@200 cd/m^2^	2019 [40]
ZnSeTe/ZnSeS/ZnS	ITO/PEDOT:PSS/TFB:PVK/QDs/ZnMgO/Al	455	40	6	0.33	261	—	2020 [77]
ZnSeTe/ZnSe/ZnSeS/ZnS	ITO/PEDOT:PSS/PVK/QDs/ZnMgO@Mg(OH)_2_/Al	447	28	5.5	9.5	2,904	—	2020 [81]
ZnSeTe/ZnSe/ZnS	ITO/PEDOT:PSS/PVK/QDs/TmPyPB/LiF/Al	450	26	5.13	4.06	3,200	—	2020 [88]
ZnSeTe/ZnSe/ZnS	ITO/PEDOT:PSS/TFB/QDs/ZnMgO/Al	460	36	2.6	20.2	88 900	15,850 h	2020 [49]
ZnSeTe/ZnSe/ZnS	ITO/PEDOT:PSS/PVK/QDs/ZnMgO/Al	458	—	—	18.6	10 398	—	2022 [66]
ZnSeTe/ZnSe/ZnS	Ag/IZO(PTL)/ZnMgO/QDs/CBP/MoO_3_/IZO/SiO_2_	476	54.2	—	18.16	—	—	2022 [89]
ZnSeTe/ZnSe/ZnS	ITO/PEDOT: PSS/PVK/QDs/ZnMgO/Al	445	—	5.9	5.46	332	93 s@120 cd/m^2^	2022 [73]
ZnSeTe/ZnSe/ZnS	ITO/PEDOT:PSS/TFB/QDs/ZnMgO/Al	452	22	3.3	18	3,520	49.1 h	2023 [67]
ZnSeTe/ZnSe/ZnS	Ag/ITO/HIL/HTL/QDs/ZnMgO/Mg:Ag/CPL	460	—	—	5.7		—	2023 [90]
ZnSeTe/ZnSe/ZnS	ITO/PEDOT:PSS:PFI/PVK/QDs/ZnMgO/Al	465	25	—	4	1,500	—	2023 [58]
ZnSeTe/ZnSe/ZnS	ITO/PEDOT:PSS/TFB/QDs/ZnMgO/Ag	448	—	3.0	10.9	10 240	—	2024 [68]
ZnSeTe/ZnSe/ZnS	ITO/PEDOT:PSS/TFB/QDs/ZnMgO/Al	460	30		14.7	3,516	52.1 h	2024 [76]
ZnSeTe/ZnSe/ZnS	ITO/PEDOT:PSS/TFB/QDs/ZnMgO/Al	462	—	2.6	17.2	13 677	—	2024 [62]
ZnSeTe/ZnSe/ZnS	ITO/PEDOT:PSS/TFB/QDs/ZnMgO/Al	456	—	2.5	5.6	14 125	1,301 h	2024 [84]
ZnSeTe/ZnSe/ZnS	ITO/PEDOT:PSS/TFB/QD/ZMO/Al	—	—	—	17.1	39 739	8,224 h	2024 [83]
ZnSeTe/ZnSe/ZnS	ITO/PEDOT:PSS/PVK/QD/CBP/MoO_3_/Al	446	—	5.1	2.25	710	—	2024 [72]
ZnSeTe/ZnSe/ZnS	ITO/PEDOT:PSS/PVK/QDs/ZnMgO/Al	454	—	4.6	9.1	6,370	—	2024 [74]
ZnSeTe/ZnSe/ZnS	ITO/PEDOT:PSS/PF8Cz/QD/POT_2_T/ZMO‐POT_2_T/Al	458	—	3.4	18.02	44,037	618 h	2025 [85]
ZnSeTe/ZnSe/ZnS	ITO/PEDOT:PSS/PF8Cz/QDs/ZMO‐Cl@Mg(OH)2/Al	456	32	2.4	17.5	13,670	5.1 h@1,000 cd/m^2^	2025 [82]
ZnSeTeS/ZnSe/ZnS	ITO/PEDOT:PSS/PF8Cz/QDs/ZMO/Al	460	17	3.0	24.7	36,850	29 600 h	2025 [60]

## 6 Summary and Outlook

This review provides a comprehensive review of recent advancements in Zn‐based blue‐emitting QDs and their application in QLEDs. Due to its wide bandgap, ZnSe QDs typically exhibit emission in the blue‐violet region. When passivated with a ZnS shell, the QDs achieve high PLQY, enabling QLED devices to attain an EQE exceeding 10%. However, the emission wavelength remains shorter than optimal for commercial display applications. Although enlarging the QD size can redshift the PL peak beyond 450 nm, this strategy often leads to a significant deterioration in EL performance, presenting a major challenge for device optimization. By doping Te into ZnSe to form ZnSe_x_Te_1‐x_ QDs, the bandgap can be effectively reduced, shifting the PL peak into the desired blue region. Precise control over the thickness of the ZnSe/ZnS double shell, combined with advanced surface ligand engineering, further enhances the PLQY to nearly 100%. The combined optimization of QLED device structure and charge transport layers has enabled an EQE exceeding 20%. However, due to the significant difference in ionic radii between Te and Se, the emission spectrum exhibits noticeable broadening in the long‐wavelength region. This phenomenon is likely attributable to hole localization caused by Te ion clustering. Recent studies have demonstrated that incorporating highly electronegative S atoms effectively suppresses the formation of Te clustering centers, thereby reducing the FWHM. As a result, the PLQY can reach up to 100%, an LED device exhibiting an EQE of 24.7% has been successfully fabricated, and the T_50_ operational lifetime reaches 29 600 h at 100 cd/m^2^, representing the best EL properties reported to date for Cd‐free blue‐emitting QDs.

Blue ZnSeTe QDs have undergone rapid development, achieving performance levels comparable to those of Cd‐based QDs within less than a decade. However, their stability parameters still fall to meet the requirements for commercial display applications. Moreover, the majority of laboratory‐scale research has been confined to conventional device structure, with limited investigation into top‐emission device that are critical for display panel integration. Pixilation techniques remain entirely unexplored. Future research efforts should prioritize advancements in these key areas. The following recommendations are proposed for consideration:

- The synthesis of ZnSe(Te) quantum dots imposes strict environmental requirements. The diverse precursors (including Se‐TOP, Se‐DPP) utilized are extremely sensitive to water and oxygen in the air, consequently leading to a comparatively high preparation cost. The further development of more appropriate preparation schemes and more stable passivation strategies holds great significance for enhancing the reproducibility of QDs preparation and reducing costs.
- The operational stability of QLED devices is currently inadequate for commercialization, representing a major obstacle to the development of full‐color QLED displays. Owing to the wide bandgap of blue‐emitting QDs, higher operating voltages are necessitated, resulting to enhanced heat generation during device operation. This places higher demands on the thermal stability of all functional layers. To enhance device robustness, improved material design is crucial—utilizing ligands with superior thermal stability to passivate the EML can significantly improve its thermal resilience. Furthermore, the conventional ZnMgO ETL may benefit from integration a protective shell structure, which helps maintain the structural integrity and morphological stability of nanoparticles under operational stress.
- The development of novel transport layer materials for blue‐emitting QLEDs is significant. Owing to the large bandgap of blue‐emitting materials, the valence band is situated at a deeper energy level. However, the majority of currently available HTL materials possess HOMO energy levels that are comparatively shallow, hereby creating a considerable energy barrier at the interface between the HTL and the EML. This barrier hinders efficient hole injection, resulting in an imbalance in carrier injection. Hence, the rational design of HTL materials with deeper HOMO energy levels is essential to facilitate hole injection and achieve balanced carrier transport. Moreover, inorganic HTL materials may exhibit enhanced stability relative to organic alternatives, as their well‐defined crystalline structures generally demonstrate superior thermal and environmental stability compared to amorphous polymer layers.
- Currently, various printing and coating techniques for QD solutions have advanced to a relatively mature stage, with preliminary progress also achieved in pixelation processes. Photolithography is widely utilized for patterning the EML. However, to date, no studies have been reported on the pixilation of Zn‐based QDs. It remains uncertain whether the lithographic processes applied to this material system may compromise the integrity of the QDs or negatively impact their luminescent performance. In‐depth research in this area represents a critical step toward the commercialization of QLED devices.

Blue‐emitting Zn‐based QDs have demonstrated outstanding performance in replacing Cd‐based QDs, showcasing rapid advancements and considerable potential for practical applications. With continuous research progress and technological innovations, the commercialization of Zn‐based blue‐emitting LEDs is steadily progressing, thereby bringing next‐generation display technologies closer to realization.

## Conflicts of Interest

The authors declare no conflicts of interest.

## Data Availability

The authors have nothing to report.
